# Trophic lengthening triggered by filamentous, N_2_ ‐fixing cyanobacteria disrupts pelagic but not benthic food webs in a large estuarine ecosystem

**DOI:** 10.1002/ece3.11048

**Published:** 2024-02-20

**Authors:** Markus Steinkopf, Uwe Krumme, Detlef Schulz‐Bull, Dirk Wodarg, Natalie Loick‐Wilde

**Affiliations:** ^1^ Department of Biological Oceanography Leibniz Institute for Baltic Sea Research Warnemuende Rostock Germany; ^2^ Thünen Institute of Baltic Sea Fisheries Rostock Germany; ^3^ Department of Marine Chemistry Leibniz Institute for Baltic Sea Research Warnemuende Rostock Germany

**Keywords:** Baltic Sea, cod, compound‐specific nitrogen stable isotopes, cyanobacterial blooms, eutrophication, flounder, food web, trophic lengthening

## Abstract

Eutrophication, increased temperatures and stratification can lead to massive, filamentous, N_2_‐fixing cyanobacterial (FNC) blooms in coastal ecosystems with largely unresolved consequences for the mass and energy supply in food webs. Mesozooplankton adapt to not top‐down controlled FNC blooms by switching diets from phytoplankton to microzooplankton, resulting in a directly quantifiable increase in its trophic position (TP) from 2.0 to as high as 3.0. If this process in mesozooplankton, we call trophic lengthening, was transferred to higher trophic levels of a food web, a loss of energy could result in massive declines of fish biomass. We used compound‐specific nitrogen stable isotope data of amino acids (CSIA) to estimate and compare the nitrogen (N) sources and TPs of cod and flounder from FNC bloom influence areas (central Baltic Sea) and areas without it (western Baltic Sea). We tested if FNC‐triggered trophic lengthening in mesozooplankton is carried over to fish. The TP of cod from the western Baltic (4.1 ± 0.5), feeding mainly on decapods, was equal to reference values. Only cod from the central Baltic, mainly feeding on zooplanktivorous pelagics, had a significantly higher TP (4.6 ± 0.4), indicating a strong carry‐over effect trophic lengthening from mesozooplankton. In contrast, the TP of molluscivorous flounder, associated with the benthic food web, was unaffected by trophic lengthening and quite similar reference values of 3.2 ± 0.2 in both areas. This suggests that FNC blooms lead to a large loss of energy in zooplanktivorous but not in molluscivorous mesopredators. If FNC blooms continue to trigger the detour of energy at the base of the pelagic food web due to a massive heterotrophic microbial system, the TP of cod will not return to lower TP values and the fish stock not recover. Monitoring the TP of key species can identify fundamental changes in ecosystems and provide information for resource management.

## Graphical abstract

Trophic pyramid of pelagic and benthic food webs in the western (left) and central Baltic Sea (right) without and with impacts of filamentous N_2_‐fixing cyanobacteria (FNC) blooms and massive influence of the microbial system, respectively, including the trophic positions (TPs) of the key species measured in this study, cod and flounder. Only the pelagic food web in the central Baltic Sea is affected by the carry‐over effect of trophic lengthening (increase in TP compared to the local reference TP value and global mean TP value) at the dietary base due to the growth of FNCs and associated microzooplankton and influence of the microbial System, which together cause increased TPs in the pelagic food web up to the level of cod. In contrast, the benthic food web is largely unaffected by FNC‐triggered trophic lengthening. FNC‐triggered trophic lengthening inevitably leads to large losses of thermal and chemical energy in between primary producers and zooplankton that propagate through the pelagic food web in the central Baltic Sea.

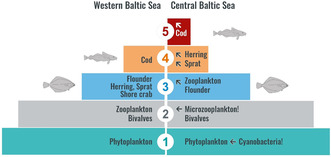

## 1 INTRODUCTION

Blooms of filamentous, N_2_‐fixing cyanobacteria (FNCs) are predicted to increase in the future due to rising sea surface temperatures, increased seasonal stratification but also due to nearshore eutrophication, namely phosphorous (Hallegraeff, 1993; Joehnk et al., 2008; Kahru et al., 2020; Paerl & Huisman, 2008; Viitasalo & Bonsdorff, 2022; Wurtsbaugh et al., 2019). Hot spots of coastal FNCs occur around the globe, for example, in the Pacific off Australia (Ani et al., 2023; Bell, 2021; Bolch et al., 1999; Hallegraeff et al., 2021), the Atlantic off the Canary Islands (Benavides & Arístegui, 2020), the southern South China Sea off Vietnam (Tang et al., 2004), the Arabian Sea off India (D'Silva et al., 2012), or the Baltic Sea (O'Neil et al., 2012) but sometimes also in the Mediterranean Sea (Rahav & Bar‐Zeev, 2017). FNCs are not inherently toxic, but they are rarely grazed upon directly. Importantly, FNCs change the phytoplankton species composition so that availability of directly palatable autotrophic species decreases in favor of a massive heterotrophic microbial system. Thus, in an ecosystem FNCs can cause a directly measurable increase in the TPs of mesozooplankton, we call trophic lengthening (Loick‐Wilde et al., 2019; Mulholland, 2007; Paerl, 1988). Since FNCs are not top‐down controlled and remain in surface waters due to their robust gas vacuoles even after microbial degradation has started (Loick‐Wilde et al., 2018; Mulholland, 2007), carnivory, rather than herbivory, dominates in FNC‐affected mesozooplankton communities, particularly during aging and decaying FNC blooms (Loick‐Wilde et al., 2019; Weber et al., 2021).

As evidence for trophic lengthening in FNC‐affected mesozooplankton communities is mounting, there is a growing need for understanding the effects on the productivity of higher trophic levels like fish. So far it is unclear, whether FNCs and the associated trophic lengthening in mesozooplankton, caused by an increasing influence of the microbial system, also affects the TPs of commercially important fish species like cod and flounder. Any increase in the TPs of fish species would have massive negative consequences for the stock productivity because trophic lengthening directly reduces the efficiency of energy transport to fish (Mulholland, 2007; Paerl, 1988; Reichle, 2023).

The TP of fish and the inorganic nitrogen source (N source) supporting fish production can now be determined empirically from different field locations using compound‐specific analysis of stable nitrogen isotopes in amino acids (CSIA). The strength of CSIA lies in providing information on both TP and N sources from a single fish sample, which is achieved with a comparison of the δ^15^N values of two different groups of amino acids (AA), the source AAs and the trophic AAs. While trophic AAs are enriched in ^15^N by ∼8.0‰ per trophic transfer (Chikaraishi et al., 2009), the δ^15^N of source AAs remain nearly unchanged when the AA is transferred through the food web and thus they reflect the isotopic composition of the primary producers (N source measure; Chikaraishi et al., 2010; McClelland & Montoya, 2002; Steffan et al., 2015). With the δ^15^N ratio of both amino acid groups and the trophic discrimination factor (TDF) that is specific for different groups of organisms, the TP of vertebrates like fish can now be calculated empirically with an accuracy of 0.1–0.2 units (Bradley et al., 2015; Brault et al., 2019; McMahon et al., 2015).

Sea areas where nitrogen from N_2_ fixation (diazotroph nitrogen) is an important new N source for biological production have in common that the isotopic signature of nitrogen in thermocline nitrate, which is the other important new N source in the ocean besides N_2_, uniquely reflects the impact of N_2_ fixation on a habitat or ecosystem (Sigman & Fripiat, 2019). This is the case, for example, in the subtropical Pacific (Casciotti et al., 2008), subtropical Atlantic (Knapp et al., 2005), or central Baltic Sea (Korth et al., 2014; Voss et al., 2005). These areas have in common that the δ^15^N value of thermocline nitrate is around 3.6‰ or lower. The strong N source proxy quality of the δ^15^N of phenylalanine and other source amino acids allow for an end‐to‐end quantification of inorganic nitrogen sources like diazotroph nitrogen or thermocline nitrate fueling new production up to the trophic position of top carnivores (Glibert et al., 2019; McMahon & McCarthy, 2016; Ohkouchi et al., 2017).

Stable isotope data, when considered alone, can lead to misinterpretation as the same isotopic value can reflect different processes (see “Fretwell's Law” in Kendall & Caldwell, 1998). To ensure that evidence from stable isotopes is useful, patterns in stable isotope data must be supported by other oceanographic, biogeochemical, and population ecology information from the ecosystem (Fry, 2006; Kendall & Caldwell, 1998). A well‐studied example and model for the biogeochemical and ecological effects of FNCs is the Baltic Sea (Elmgren, 2001; Reusch et al., 2018; Voss et al., 2005). The Baltic Sea is a postglacial, semi‐enclosed, heavily exploited, microtidal, brackish‐water sea that experiences numerous anthropogenic pressures, especially eutrophication (Conley, 2012; Voss et al., 2011). Despite high nutrient loads, during the midsummer period, the dissolved nitrate pools in surface waters are largely depleted, but not the phosphorus pools. Under these conditions, cyanobacteria are competitively superior to other algae leading to massive blooms of unpalatable FNCs that frequently occur in the central Baltic Sea (Kahru et al., 2020; Karlson et al., 2015; Wasmund, 1997). Their N_2_ fixation is the second largest source of new N for the Baltic Sea, adding 370–926 kt N year^−1^ into the central Baltic Sea during summer (Voss et al., 2005). The largest new N source for the Baltic Sea is anthropogenic N, for example, from terrestrial run‐off via rivers like the Odra or Vistula. Rivers transport on average 830 kt N year^−1^, mainly as nitrate, into coastal waters around the entire Baltic and the relatively shallow western Baltic Sea, especially in winter and spring (Kuliński et al., 2022; Reckermann et al., 2022; Voss et al., 2005). The spatial and temporal dichotomy of N_2_ fixation and anthropogenic N inputs between the central and western Baltic Sea go along with the other contrasting ecological and biogeochemical differences, which are highly relevant to understand the dynamics in the trophic position and nitrogen supply of adult fish species in the two areas of the Baltic Sea:

1. Phytoplankton quality: Unpalatable FNCs dominate the new production in the central Baltic Sea in summer (*Nodularia spumigena*, *Aphanizomenon* sp.), whereas palatable phytoplankton like diatoms (namely *Cerataulina bergonii, Rhizosolenia* spp., *Skeletonema marinoi*) and dinoflagellates dominate new production in the western Baltic Sea during spring and fall (Dutz et al., 2022; Neumann, 2010; Wasmund et al., 2011; Zettler et al., 2020).
2. TP of mesozooplankton: Omni‐ and carnivorous mesozooplankton with a TP of 2.5 to 3.0 dominates in the central Baltic Sea due to unpalatable FNC blooms and the associated massive heterotrophic microbial system, while herbivorous mesozooplankton with a TP of 2.0 dominates in the western Baltic Sea and nearshore areas given that the phytoplankton is palatable (Loick‐Wilde et al., 2019).
3. δ^15^N values in water‐column‐nitrate and sediments: In the central Baltic Sea, N_2_‐fixation causes low δ^15^N values of 3.6 ± 1.0‰ in thermocline nitrate and surface sediment particles. In the western Baltic and coastal areas, anthropogenic N causes high δ^15^N values of 7.2 ± 0.9‰ to 7.9 ± 1.8‰ in thermocline nitrate and surface sediment particles (Korth et al., 2014; Voss et al., 2005). Here we used the δ^15^N of the source amino acids in samples from adult cod and flounder to dedicate the samples to a habitat either largely influenced by N_2_ fixation or inputs of anthropogenic N. Specifically, the δ^15^N of source amino acids were used to identify how tightly the fish are associated with a thermocline nitrate pool that is largely influenced by either of the two N sources (N_2_ or anthropogenic N).

Altogether, this makes the Baltic Sea an ideal model ecosystem to test the hypothesis that trophic lengthening in mesozooplankton due to a massive heterotrophic microbial system triggered by FNCs is transferred also to organisms from higher trophic levels like fish that are associated with the pelagic and benthic food webs.

To verify this hypothesis, our analysis focuses on individuals of cod and flounder from populations in the western and central Baltic Sea. Both species rely on new production of phytoplankton for growth, but depending on the location of their population, they rely on very different food webs to receive the energy. In the Baltic, flounder mainly use shallow coastal areas and only migrate into deeper waters for spawning in the first quarter of the year. They use relatively small foraging areas (e.g., Dando, 2011) and mainly feed on bivalves (Haase et al., 2020). Since bivalves unselectively filter‐feed on any sedimenting phytoplankton, including cyanobacteria, flounders are unlikely to be affected by FNC‐triggered trophic lengthening in more selectively feeding mesozooplankton, as most of the emerging microbial food web around FNCs will not sink down as the FNCs do after some time (Mulholland, 2007). Therefore, this key part of trophic lengthening should not appear in the benthic food web. Cod is a flexible predator preying on invertebrates and fish and can move large distances. In the western Baltic, the cod diet is dominated by the common shore crab *Carcinus maenas* (Funk et al., 2021). In contrast, cod in the central Baltic Sea mainly feed on pelagic fishes like *zooplanktivor*ous sprat and herring (Haase et al., 2020; Kulatska et al., 2019). It is likely that cod from the central Baltic Sea display a higher TP than cod from the western Baltic due to both, different foraging strategies and trophic lengthening in mesozooplankton triggered by FNCs, as both processes are limited to the central Baltic (Loick‐Wilde et al., 2019).

Here, for the first time, we can distinguish between spatial and species‐specific changes in the inorganic N source supporting the fish production in relation to changes in trophic structure in pelagic and benthic food webs in the Baltic Sea. We use δ^15^N‐AA measurements from samples of demersal fish species differently associated with the pelagic and benthic food webs in their areas to resolve a major issue concerning the impact of trophic lengthening triggered by FNCs on marine and estuarine food webs.

## 2 MATERIALS AND METHODS

### 2.1 Study areas and fish species

**FIGURE 1:**
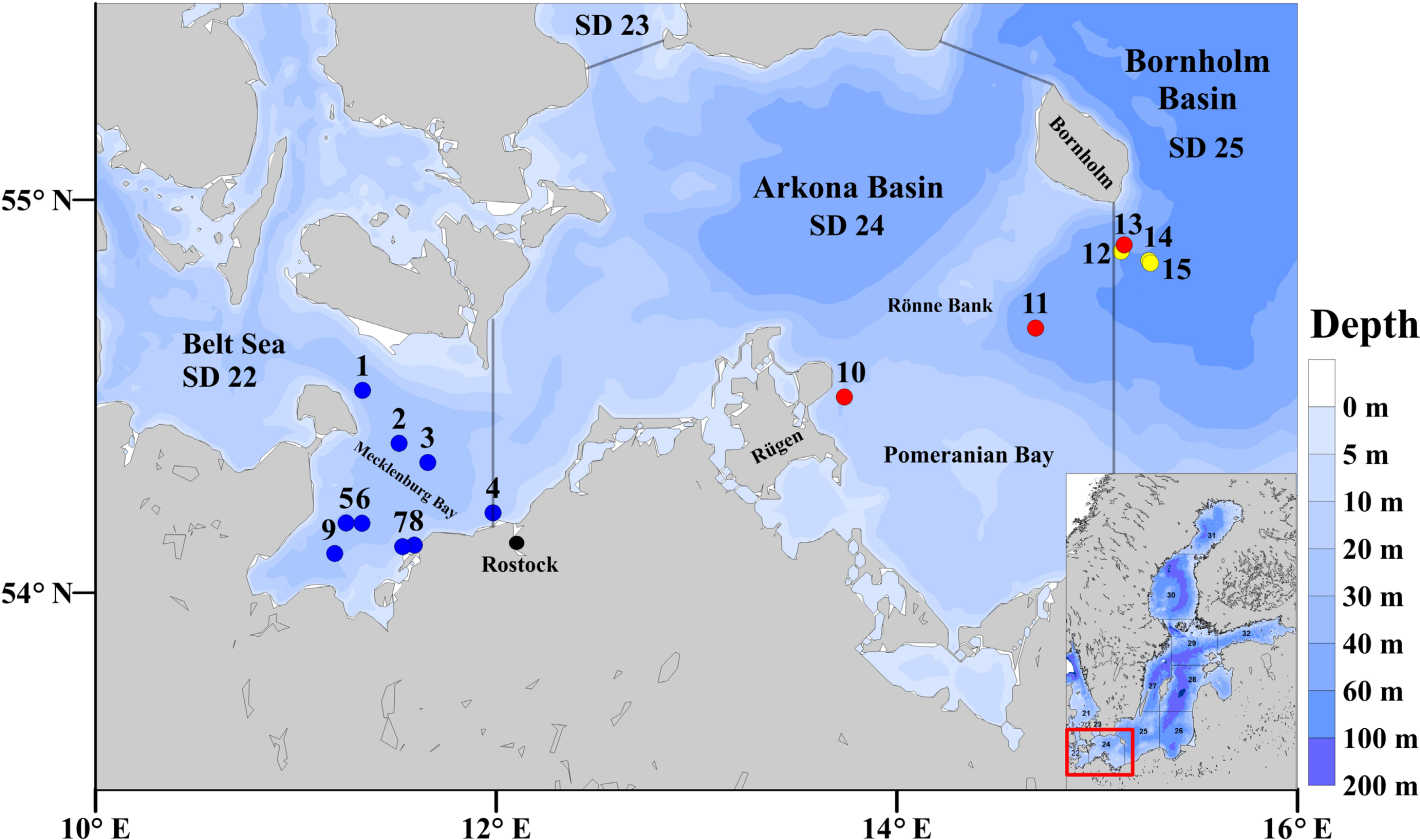
Sampling stations for Baltic cod and flounder in the western (blue) and central (red and yellow) Baltic Sea. Western Baltic Sea stations were located in the Mecklenburg Bay as part of the Belt Sea in 2020. Central Baltic Sea stations were located near Rügen and the south‐western Bornholm Basins in 2019 (red) and 2020 (yellow). Inlay map shows the entire Baltic Sea.

The study was conducted in an area with no or minor impact of FNCs, the western Baltic Sea, and an area regularly affected by FNCs, the central Baltic Sea (Wasmund et al., 2011, Figure 1). Fish from the western Baltic Sea were sampled specifically in the Mecklenburg Bay, which is part of the Belt Sea. 98% of the Belt Sea is shallower than 30 m. Wind‐driven hydrodynamics interact with changes in the two‐layer exchange flow of more saline bottom water from the Kattegat and surface outflow from the central Baltic Sea through the Danish Straits. Salinities are relatively high compared to the central Baltic Sea. Fish from the central Baltic Sea were sampled in the southern Bornholm Basin, except for one station near Rügen (Figure 1). Water depth of 100 m, permanent stratification and extensive oxygen minimum zones in the basin characterize the area.

Baltic cod (*Gadus morhua*) and European flounder (*Platichthys flesus*) are two of the most important commercially used demersal fish species in the Baltic Sea and play a major role in the benthic and pelagic food webs. Due to their significance in the Baltic Sea and distinct feeding habits, these two fish species were chosen for this study.

### 2.2 Fish muscle tissue sample collection

The fishes originated from four cruises to the western Baltic Sea (ICES subdivision (SD) 22) and central Baltic Sea (SD 24–25) in January/February 2019 and 2020 (Figure 1, Table S1). In the management area SD 24–25 offshore spawning European flounder *P. flesus* and the coastal spawning Baltic flounder *Platichthys solemdali* (a recently described new species; Momigliano et al., 2018) are present, with an estimated share (from survey data of 2014 and 2015) of approximately 85% and 15%, respectively (ICES, 2023). Presently, species assignment is only possible using genetics. Here, we assume that all flounder were *P. flesus*.

The fish were caught by bottom trawl, with a Bacoma cod end (2019) and a TV30 #520 trawl gear (2020) following the standards of the Baltic International Trawl Survey (ICES, 2017). On board, the fish were killed following German regulations, identified to species level, counted, and measured. The otoliths were extracted and a sample of white muscle tissue was taken from behind the third dorsal fin of 30 cod individuals and from the tail muscle of 21 flounder individuals. All samples were frozen immediately at −20°C for later stable isotope analyses. All fishes were sampled within a certain size range (cod: 20–40 cm, flounder 20–30 cm), to ensure comparability between the two areas and be sure that there is no major length or age effect in the trophic position calculations.

Cod and flounder otoliths were embedded, sliced and aged using the standard procedures for the two species in the Baltic Sea region. Age determination revealed an age range for cod (median length 30.5 ± 5.0 cm) of 1–4 years (median: 2 years) and for flounder (median length 25.0 ± 5.0 cm) from 2–4 years (median: 3 years), respectively (Table S1).

Previous stock assignment of cod otoliths from the two areas using otolith‐shape analyses (Schade et al., 2019, 2022) revealed a ratio of western to eastern Baltic cod of 100:0 and 35:65 in SD 22 and SD 24–25, respectively, and may apply to some extent also to our sample set of cod (Table S1).

### 2.3 Sample preparation and derivatization

The frozen cod und flounder muscle tissue samples were cleaned mechanically and with distilled water to remove surface contaminants, freeze dried (Christ Alpha 1–4), and then ground and homogenized for further processing. For CSIA, ~10 mg of each dried sample was transferred into a heat‐resistant borosilicate vial, mixed with 5 mL of 6 M HCl solution and 1 mL of internal standard (trans‐4 (amino methyl)‐cyclohexane carboxylic acid), and hydrolyzed for 24 h at 110°C. The samples were then filtered through cellulose‐acetate filters, dried under a nitrogen flow at 50°C, and then derivatized to TFA‐isopropyl amino esters (Hofmann et al., 2003; Silfer et al., 1991), which included an additional purification step using a chloroform‐phosphate buffer solution (Veuger et al., 2005). The derivatized samples were dissolved in 500 μL of methylene chloride and stored in GC‐vials at −20°C until analyzed as described below.

### 2.4 Bulk and compound‐specific isotope measurements

The measurements of the bulk δ^13^C of the muscle tissue samples were carried out according to Loick et al. (2007), using Elemental Analyzer Isotope Ratio Mass Spectrometry (EA‐IRMS) analyses of the fish muscle tissue samples (Thermo Finnigan Delta Plus + Thermo Flash EA 1112). For this purpose, the dried and powdered fish muscle tissue samples were weighed (~0.5 mg per sample), packed in tin boats and measured by EA‐IRMS. Calibration for the total carbon determination was done daily with an acetanilide standard (Merck). All isotope abundances are expressed in δ notation as follows: δ X (‰) = [(*R*_sample_/*R*_standard_) – 1] * 10^3^, where X is ^13^C, and *R* is the ^13^C:^12^C ratio. The internal laboratory reference gas for the C‐analyses was ultrapure CO_2_, which was calibrated against the materials from the International Atomic Energy Agency (IAEA): NBS 22 (mineral oil δ^13^C = −29.74 ‰) and USGS 24 (graphite δ^13^C = −15.99 ‰). In addition, peptones (Merck) were analyzed as in‐house standards after every sixth fish sample run. The analytical error for stable isotope ratios indicated by the peptone standards was less than ±0.2 ‰ for carbon isotopes.

The TFA‐isopropyl‐derivatized samples were analyzed for their content of δ^15^N in 13 AAs; an external standard (16 AAs) was also included. The 13 AAs were: alanine (Ala), glycine (Gly), threonine (Thr), serine (Ser), valine (Val), leucine (Leu), isoleucine (Ile), proline (Pro), aspartic acid (Asp), glutamic acid (Glu), phenylalanine (Phe), tyrosine (Tyr), and lysine (Lys). The concentrations of cysteine (Cys), arginine (Arg), and methionine (Met) in the samples were below the qualitative detection limit of the measurement device and could, therefore, not be determined in the fish muscle tissue samples. During the initial hydrolysis step, glutamine and asparagine were converted into glutamic acid and aspartic acid, respectively, such that they were considered together as Glu + Gln (referred to herein as Glu) and Asp + Asn (Asp; Brault et al., 2019).

Amino acid‐specific δ^15^N values were measured using an isotope ratio mass spectrometer (IRMS, Thermo Finnigan GmbH, MAT 253 MS, Germany) connected via a ConFlo IV interface unit to a gas chromatograph combustion periphery (GC‐C, Thermo Scientific Trace 1310 GC, Italy; Thermo Scientific, GC Isolink, Germany). The separation column in the GC consisted of a non‐polar column coated with 5% phenyl‐polysilphenylenesiloxane (BPX5, 60 m, 0.32 mm inner diameter, film thickness of 1 μm; SEG Analytical Science, Ringwood, Victoria, Australia). For each run, 2 μL of sample was injected via a PTV injector in splitless mode. The temperature program was as follows: Initial temperature at 50°C, heat up at 12°C/min to 120°C, hold for 17 min, heat up at 3°C/min to 180°C, linger for 10 min, heat up at 5°C/min to 200°C, hold for 6 min, heat up to 250°C at 10°C/min and hold for 7 min. Each sample was injected and measured at least three times using helium (He) as the carrier gas. The standard deviation from three runs was usually <1.0 ‰ for all 13 AAs.

### 2.5 Bulk δ^13^C values in cod and flounder as migratory marker

We used the relationship between sea surface salinity and the non‐lipid corrected δ^13^C values of total organic carbon in cod and flounder as marker for the level of exchange of the fish between the western and central Baltic Sea (Larsen et al., 2020).

### 2.6 Best fitting TP model to estimate the TP of cod and flounder

There are currently four CSIA‐based TP models available that are suitable to estimate the TP of vertebrates (Bradley et al., 2015; Brault et al., 2019; Germain et al., 2013; Nielsen et al., 2015). They were tested and partly modified as described below to identify, which TP model is most suitable for cod and flounder, respectively. Specifically, CSIA data of cod and flounder from the western Baltic Sea were chosen to validate and calibrate the CSIA‐based TP models based on the assumption that the CSIA‐based TPs at this reference site should be close to bulk stable isotope analysis (SIA) based TP estimates from the western Baltic Sea and ideally also close to the global mean TP value for cod of 4.1 ± 0.2 and for flounder of 3.1 ± 0.2 from Fishbase.com (Froese & Pauly, 2022). The rational for this is that the TP of cod and flounder from the western Baltic Sea should not be largely impacted by any trophic lengthening in the mesozooplankton compartment (Loick‐Wilde et al., 2019), since filamentous, N_2_‐fixing cyanobacteria and the massive microbial system triggered by them are largely absent in the western Baltic Sea. The SIA‐based TP values for cod and flounder from the reference site were calculated based on bulk δ^15^N data from literature (Mittermayr et al., 2014; Mohm, 2014, 2018) after Post (2002) as follows:

(1) $${\mathrm{TP}}_{\text{Bulk}}=\gamma +\frac{\delta^{15}N_{\text{higher consumer}}-\delta^{15}N_{\text{base}}}{{∆}_{n}}$$

where *γ* is the TP of the benthic or planktonic primary consumer (e.g., *γ* = 2.0 in zooplankton in the western Baltic Sea or *γ* = 2.7 in zooplankton in the central Baltic Sea), δ^15^N_base_ is the measured value of the benthic or planktonic primary consumer, δ^15^N_higher consumer_ is the measured value of the benthic or planktonic higher consumer (e.g., shore crab, herring, sprat, cod) and *Δ*_*n*_ is the enrichment in δ^15^N of 3.4‰ per trophic level (Post, 2002).

The weighted mean values of the groups of source and trophic AAs (Equation 2) and their errors (Equation 3) were calculated as follows:

(2) $${\mathrm{AA}}_{\text{wmean}}=\frac{\sum_{i}^{n}p_{i}\times \delta^{15}N_{i}}{\sum_{i}^{n}p_{i}}$$

(3) $$\sigma =\pm \frac{1}{\sqrt{\sum_{i}p_{i}}}$$

where *p* is the weighting factor based on standard deviation of three injections per AA, and *σ* is the error for the weighted mean values for the group of AAs.

For testing the different TP models, different sets of trophic and source AA δ^15^N data (Table 1) of the cod and flounder samples from the western and central Baltic Sea were added either into Equation 4 according to Brault et al. (2019), Bradley et al. (2015), and Nielsen et al. (2015) or into Equation 5 according to Germain et al. (2013):

(4) $${\mathrm{TP}}_{\mathrm{wmn}}=\left[\frac{\left(\delta^{15}N_{\mathrm{wmt}}-\delta^{15}N_{\mathrm{wms}}-\beta_{\frac{\mathrm{Trp}}{\mathrm{Src}}}\right)}{{\mathrm{TDF}}_{\mathrm{AA}}}\right]+1$$

(5) $${\mathrm{TP}}_{\text{multi}-\mathrm{TDF}}=\left[\frac{\left(\delta^{15}N_{\mathrm{wmt}}-\delta^{15}N_{\mathrm{wms}}-{\mathrm{TDF}}_{\text{fish}}\right)}{{\mathrm{TDF}}_{\text{plankton}}}\right]+2$$

**TABLE 1:** Summary of the five TP models tested for calculating the TPs of cod and flounder.

Model ID	TP‐equation	Wms	Wmt	β_Src/Trp_	TDF plankton	TDF_AA_	TDF_Fish_	Constant	Reference
Brault	1	Phe	Glu	3.1		4.5		1	Brault et al. (2019)
		Ser	Ala						
		Gly	Ile						
Germain‐modified	2	Phe	Glu		7.6		**5.7**	2	Germain et al. (2013)
Bradley	1	Phe	Glu	3.6		5.7		1	Bradley et al. (2015)
		Ser	Ala,						
		Gly	Ile						
Nielsen	1	Phe	Glu	2.9		5.9		1	Nielsen et al. (2015)
		Ser	Ala						
		Gly	Ile						
Brault‐modified	1	Phe	Glu	3.1		4.5		1	This study
		Ser	Ala						
		Gly	**Pro**						

*Note*: wms and wmt are the different amino acids (AAs) used for the calculation of the weighted mean δ^15^N values of the trophic (wmt) and source (wms) AA, β_Src/Trp_ is the difference between the δ^15^N values of the trophic and source AA for primary producers (in ‰), TDF_AA_, TDF_fish_, and TDF_plankton_ (all in ‰) are the trophic discrimination factors which give the average δ^15^N increases in trophic AA relative to source AA per trophic level. Highlighted in bold are variables that were modified from the original model.

The δ^15^N_wmt_ and δ^15^N_wms_ are the δ^15^N values of the model‐specific trophic and source AAs. β_Src/Trp_ represents the difference between the δ^15^N values of the trophic and source AAs for primary producers (TP = 1.0). TDF_AA_, TDF_fish_, and TDF_plankton_ are the average δ^15^N increases in trophic AAs relative to source AAs per trophic level (Glibert et al., 2019; McMahon et al., 2015). Instead of the TDF value for seals (also called TEF_Seal_ in the reference) in the model of Germain et al. (2013), the TDF_AA_ value for teleost fish from Bradley et al. (2015) was applied; therefore, we refer to this model as Germain‐modified (Table 1). Further, we added a modified TP model after Brault et al. (2019), for which the trophic AA Ile was exchanged by the trophic AA Pro; therefore, we refer to this model as Brault‐modified (Table 1). The variables for all TP models are summarized in Table. 1. The standard error (SE) in the TP estimations, computed by propagation of the analytical error in the individual amino acid determinations, typically did not exceed 0.2 TP.

**FIGURE 2:**
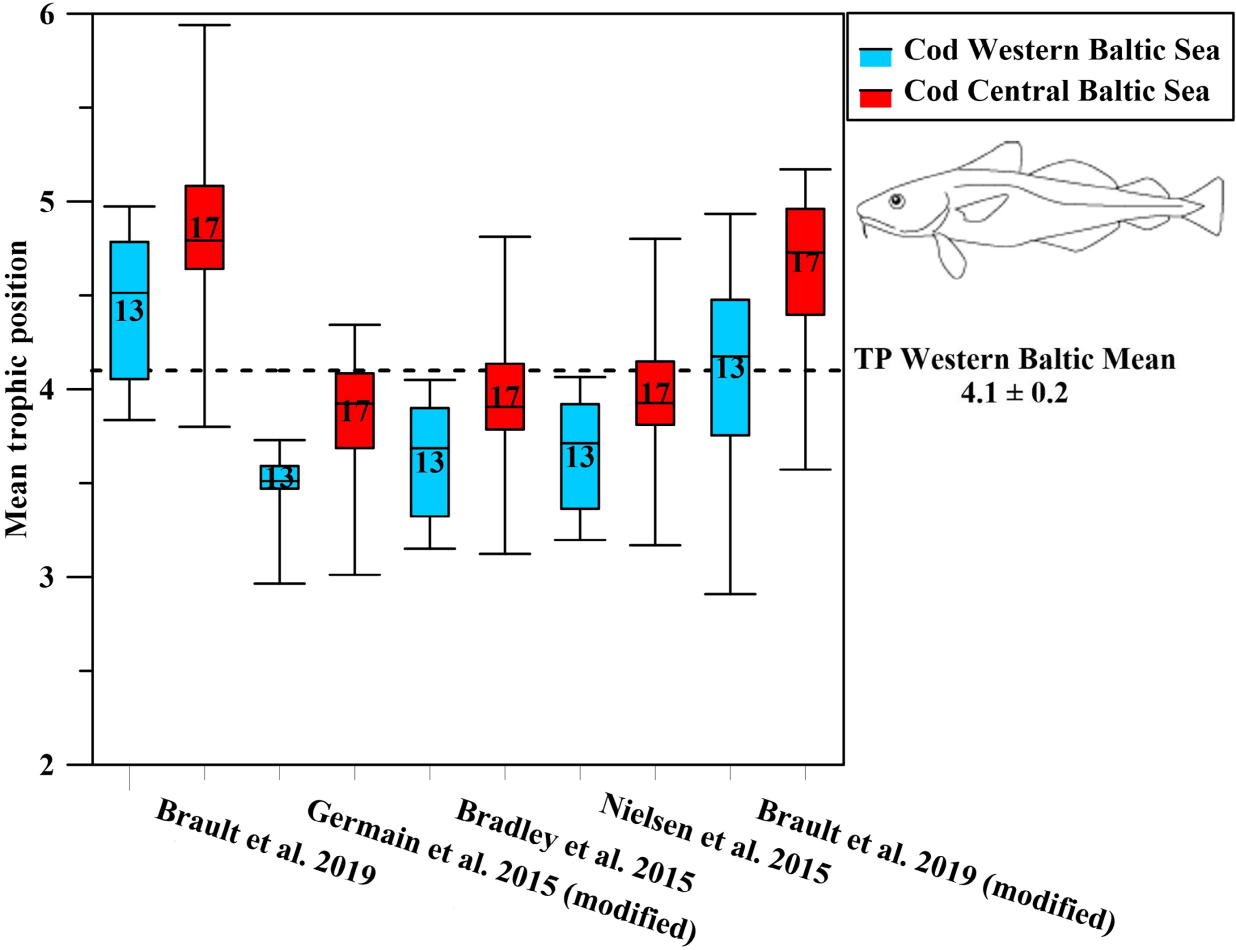
Calculated mean TP of cod from the western Baltic Sea (in blue, *n* = 13) and the central Baltic Sea (in red, *n* = 17) according to five different CSIA‐based TP models for vertebrates. The western Baltic mean TP of cod (dotted line) is based on calculated TP values based on SIA data from literature (Mittermayr et al., 2014; Mohm, 2014, 2018, Table 2).

For cod, exclusion criterion for any of the respective TP models was an extreme drop in the calculated TP values below a TP of 4.0 and towards a TP of 3.0 based on CSIA data of cod from the western Baltic Sea. Cod in the western Baltic Sea cannot reach TP values around or below 3.0, as their food base (from stomach content analyses) consisted at least of carnivorous animals (Funk et al., 2021), which makes cod a secondary carnivore and a TP below 3.0 is thus not realistic. This criterion resulted in the exclusion of the TP models after Germain et al. (2013), a modified version of Bradley et al. (2015), and Nielsen et al. (2015), while the TP model of Brault et al. (2019) yielded a mean cod TP value closest to the western Baltic mean TP of 4.1 ± 0.2 (Figure 2). We then further improved the TP result after Brault et al. (2019) for cod from the western Baltic to become congruent with the western Baltic mean TP value for cod (Figure 2, Table 2), based on calculated TP values from literature SIA data as well as stomach content analyses and model data from fischbase (Froese & Pauly, 2022; Mittermayr et al., 2014; Mohm, 2014, 2018), by a simple exchange of the trophic AA Ile with the trophic AA Pro (Table 1). The TP model modified after Brault et al. (2019) thus resulted in the best TP estimation for cod from the western Baltic Sea 4.1 ± 0.5 (Figure 2, Table 2). Notably, independent from the model applied, the TP of cod from the central Baltic Sea was always significantly higher than the TP of cod from the western Baltic Sea.

**TABLE 2:** Averages and ranges of published bulk δ^15^N values and the calculated and published TP values of cod and their key dietary organisms from the pelagic and benthic food webs of the western and central Baltic Sea.

Site	Organisms	δ^15^N bulk [‰]	δ^15^N bulk [‰]	Number	Size class	Trophic position			δ^15^N analysis	Reference	TP
		Mean ± STDV	Range	Individuals/	cm	Pelagic	Benthic	Mixed			Theoretical
				Hauls							Pelagic
Western Baltic Sea	Copepods	6.7 ± 0.9	5.6–7.7	8 Hauls	>0.01	2.0 ± 0.2	n.a.	n.a.	CSIA	Loick‐Wilde et al. (2019)	n.a.
	Copepods	7.1 ± 1.3	6.3–8.1	1490	>0.015	2.0 ± 0	n.a.	n.a.	SIA	Mittermayr et al. (2014)	n.a.
	Mussels	8.5 ± 0.2	8.1–8.7	182	n.d.	n.a.	2.0 ± 0.3	n.a.	SIA	Mittermayr et al. (2014)	n.a.
	Shore Crab	10.5 ± 1.4	9.1–11.9	4	n.d.	n.a.	3.3 ± 0.3	n.a.	SIA	Mittermayr et al. (2014)	n.a.
	Herring	12.3 ± 1.9	8.0–16.5	20	8–20.5	3.6 ± 0.4	n.a.	n.a.	*After* Post (2002)	Mohm (2014), Mohm (2018)	n.a.
	Sprat	11.9 ± 0.8	10.6–13.0	10	6.5–13.5	3.5 ± 0.2	n.a.	n.a.	*After* Post (2002)	Mohm (2018)	n.a.
	Flounder	11.9 ± 0.8	10.9–13.2	9	20–32	n.a.	3.0 ± 0.2	n.a.	*After* Post (2002)	Mohm (2014), Mohm (2018)	n.a.
	Flounder	11.7 ± 1.1	9.27–13.2	11	20–30	n.a.	**3.4 ± 0.3**	n.a.	CSIA	This study	n.a.
	Cod	14.6 ± 0.2	14.2–14.8	3	16.5–25	4.3 ± 0.0	3.8 ± 0.0	4.0 ± 0.0	*After* Post (2002)	Mohm (2014), Mohm (2018)	n.a.
	Cod	15.1 ± 0.0	14.6–15.6	10	35–39	4.4 ± 0.0	3.9 ± 0.0	4.2 ± 0.0	*After* Post (2002)	Deutsch and Berth (2006)	n.a.
	Cod	12.7 ± 1.2	9.9–14.9	13	20–35	n.a.	n.a.	**4.1 ± 0.5**	CSIA	This study	n.a.
Central Baltic Sea	Copepods	4.2 ± 0.3	2.1–4.8	12 Hauls	>0.01	2.7 ± 0.2	n.a.	n.a.	CSIA	Loick‐Wilde et al. (2019)	2.0
	Copepods	7.1 ± 1.4	5.7–8.5	5 Hauls	>0.015	2.7 ± 0.3	n.a.	n.a.	SIA	Kiljunen et al. (2020)	n.a.
	Mussels	8.6 ± 1.9	6.7–10.5	19	n.d.	n.a.	2.0 ± 0.5	n.a.	SIA	Kiljunen et al. (2020)	n.a.
	Saduria	10.1 ± 1.4	8.7–11.5	4	n.d.	n.a.	3.0 ± 0.4	n.a.	SIA	Kiljunen et al. (2020)	n.a.
	Herring	12.3 ± 1.4	10.9–13.7	45	17.9 ± 4.1	4.0 ± 0.5	n.a.	n.a.	SIA	Kiljunen et al. (2020)	n.a.
	Sprat	11.8 ± 1.2	10.6–13.0	21	11.8 ± 1.4	4.3 ± 0.4	n.a.	n.a.	SIA	Kiljunen et al. (2020)	n.a.
	Flounder	12.8 ± 1.5	11.6–16.0	8	22–30	n.a.	3.2 ± 0.4	n.a.	*After* Post (2002)	Mohm (2014), Mohm (2018)	n.a.
	Flounder	11.4 ± 1.4	9.1–13.7	11	20–30	n.a.	**3.1 ± 0.3**	n.a.	CSIA	This study	n.a.
	Cod	13.1 ± 0.6	11.6–13.9	98	23–40	4.9 ± 0.1	4.0 ± 0.1	4.5 ± 0.1	*After* Post (2002)	Mohm (2018)	3.9 ± 0.1
	Cod	12.0 ± 0.4	11.1–12.6	10	34–39	4.6 ± 0.1	3.7 ± 0.1	4.1 ± 0.1	*After* Post (2002)	Deutsch and Berth (2006)	4.2 ± 0.1
	Cod	12.4 ± 0.8	11.2–14.4	17	20–38	n.a.	n.a.	**4.6 ± 0.4**	CSIA	This study	n.a.

*Note*: Calculation of TP values after Post (2002) on the basis of published bulk δ^15^N values for different assumed food preferences (pelagic, benthic, mixed = mix of both) and of CSIA‐based TP values from this study. For the central Baltic Sea, additional theoretical TP values for cod with a pelagic food preference were calculated excluding the effect of trophic lengthening on the mesozooplankton compartment (TP of primary consumer of 2.0 rather than 2.7). Values from this study in bold.

**FIGURE 3:**
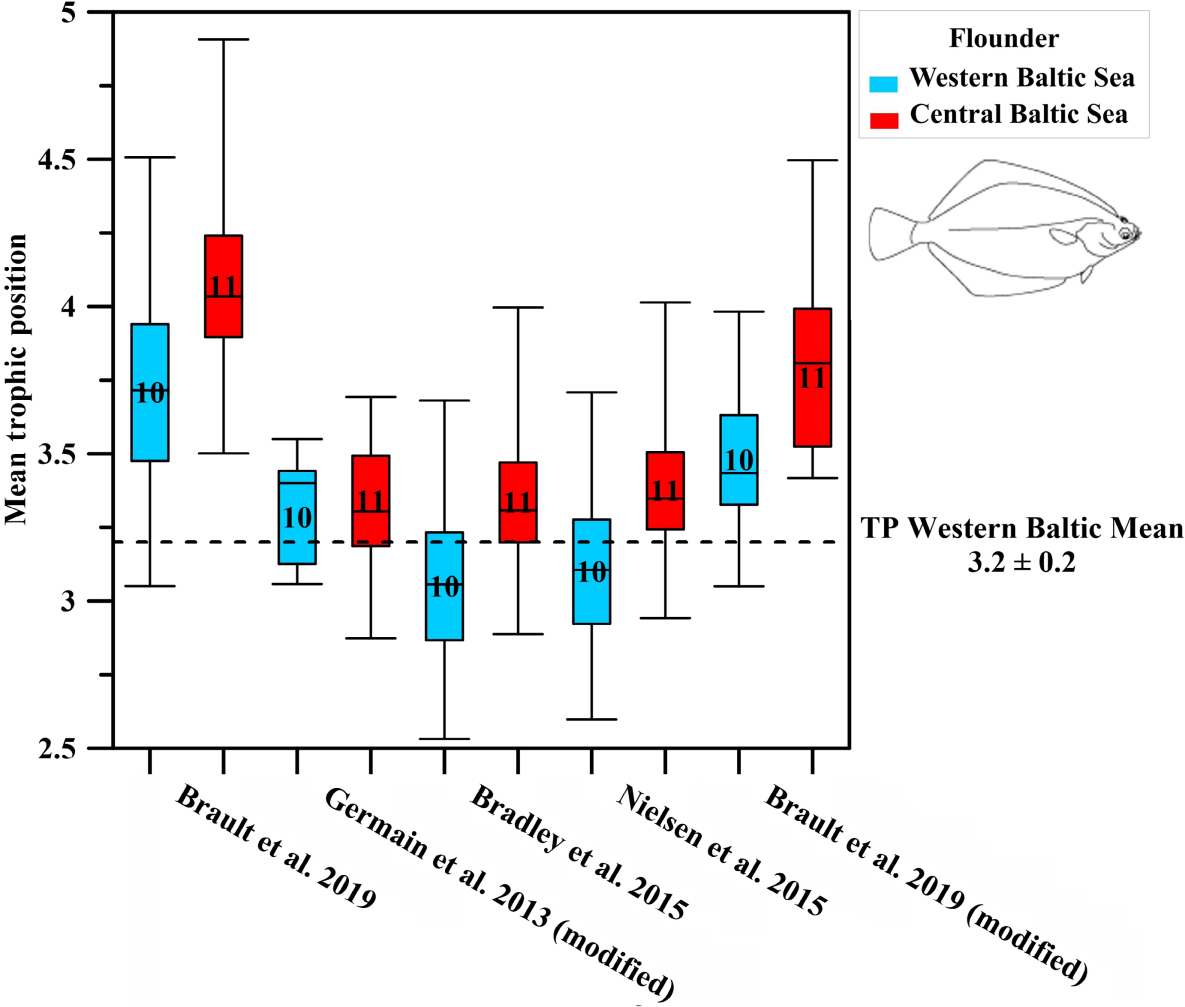
Calculated mean TP of flounder from the western Baltic Sea (in blue, *n* = 10) and the central Baltic Sea (in red, *n* = 11) according to five different CSIA‐based TP models and compared to the western Baltic mean TP of flounder based on calculated TP values based on SIA data and stomach content analysis (dotted line, Mittermayr et al., 2014; Mohm, 2014, 2018; Froese & Pauly, 2022 Table 2).

Within the flounder samples, the TP patterns calculated from the five TP models were somewhat different. The modified model after Nielsen et al. (2015), which uses the weighted mean source and trophic AA values, resulted in a mean TP value of 3.4 ± 0.3 for fish from the western Baltic Sea (Figure 3, Table 2). This value corresponds best to the western Baltic mean TP of flounder (3.2 ± 0.2) based on the calculated TPs based on SIA literature values (Mittermayr et al., 2014; Mohm, 2014, 2018) and matches also the stomach content analyses and model TP from fishbase (Froese & Pauly, 2022). All other TP models resulted in TP estimations either higher or lower than the western Baltic mean TP reference value (Figure 3). The TP model after Nielsen et al. (2015) thus resulted in the best TP estimation for flounder from the western Baltic Sea.

Interestingly, in contrast to cod no significant differences in TPs were found for most CSIA‐based TP model between flounder from the western and central Baltic Sea, except for the one after Bradley et al. (2015).

It is beyond the scope of this paper to identify why the best fitting TP estimations for cod and flounder from the western Baltic Sea compared to their reference TP mean were generated by two different TP models. However, an underlying physiological mechanism for this result may include species‐specific differences in fat and protein anabolism and catabolism (Bradley et al., 2015; Germain et al., 2013; McMahon & McCarthy, 2016).

### 2.7 Nitrogen source proxy from the best fitting TP model

The δ^15^N values of the source AAs (δ^15^N_wms_) of the cod and flounder samples from the best fitting TP model can be used as a time‐integrating proxy for the δ^15^N of the dominant inorganic nitrogen source sustaining pelagic and benthic food webs in the two areas (Loick‐Wilde et al., 2019; McClelland & Montoya, 2002; McMahon et al., 2015; Sherwood et al., 2011). In the central Baltic Sea, the high rates of N_2_ fixation by cyanobacteria result in relatively low δ^15^N endmember values in thermocline nitrate whereas in the western Baltic Sea and in coastal areas, high inputs of anthropogenic N cause much higher δ^15^N endmember values in thermocline nitrate. The consistency in the δ^15^N values between thermocline nitrate and surface sediments indicates that the spatial dichotomy between anthropogenic N and N from N_2_ fixation is robust over time scales of month to years (Korth et al., 2014; Voss et al., 2005), that are also relevant for adult fish analyzed in this study. Further, these reference values provide an index to the spatial scaling of δ^15^N in relation to the dominant N source and phytoplankton species contributing to the benthic and pelagic food webs. Hereby the low reference values of 3.6 ± 1.0‰ in thermocline nitrate and surface sediment particles from the central Baltic Sea provide a conservative estimate of the allocation to a food web strongly influenced by unpalatable, N_2_‐fixing cyanobacteria. The high reference values of 7.2 ± 0.9‰ to 7.9 ± 1.8‰ in thermocline nitrate and surface sediment particles from the Baltic southern coastal areas provide a conservative estimate of the allocation to a food web strongly influenced by palatable phytoplankton like diatoms and dinoflagellates that take up thermocline nitrate with the isotopic imprint of anthropogenic N for new production (Korth et al., 2014; Voss et al., 2005).

### 2.8 Statistical analysis

Comparisons of the western and central Baltic Sea areas across the variables were carried out using *t*‐tests or Mann–Whitney‐U‐tests and ANOVAs, depending on the variance homogeneity of the data (tested using Levene tests). All statistical analyses were carried out using R‐studio (Version 3.6.0) and the car, base, and stats packages.

## 3 RESULTS

### 3.1 Bulk δ^13^C in cod and flounder

**FIGURE 4:**
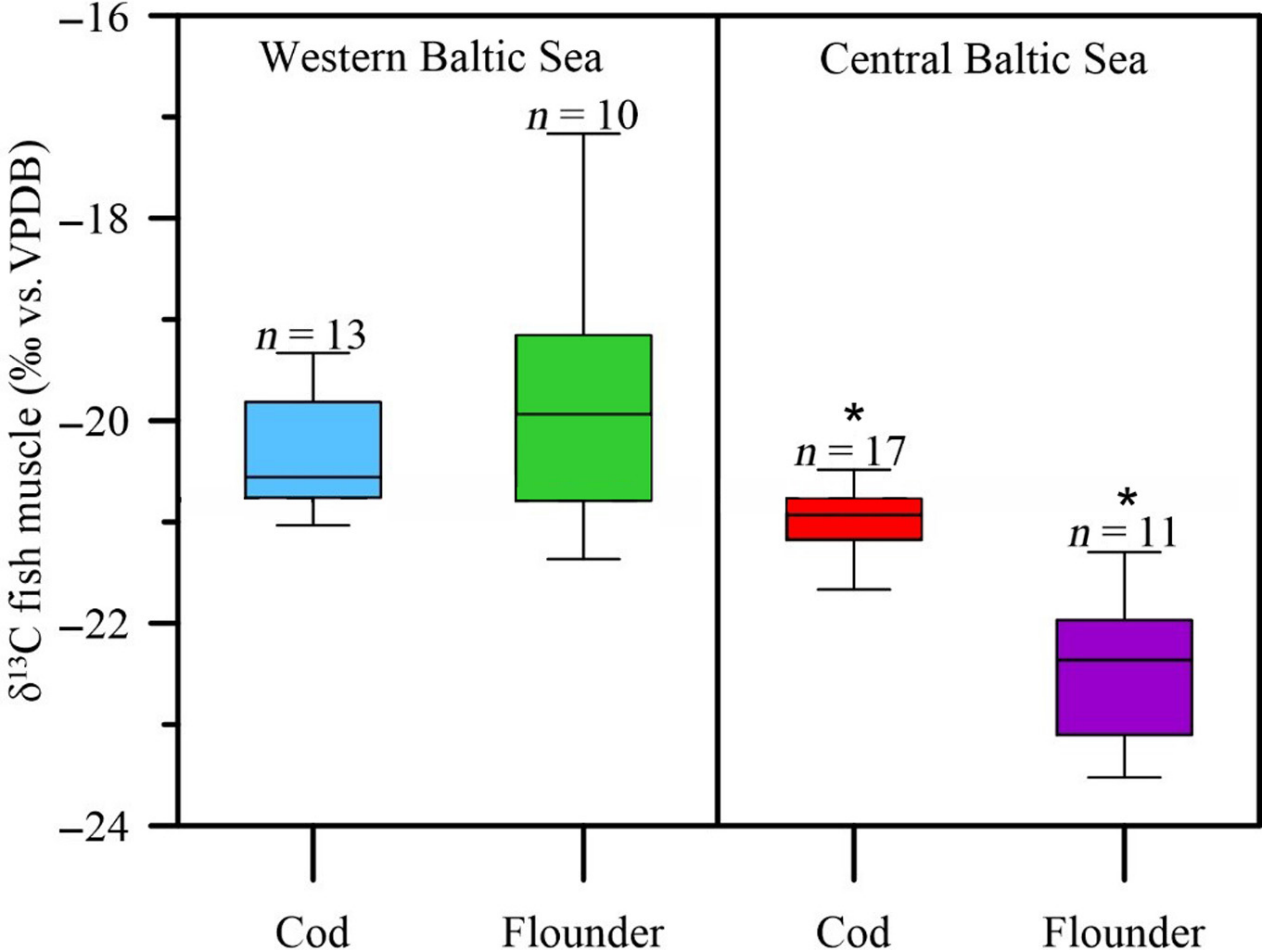
Average δ^13^C values of cod and flounder muscle tissue samples from the western and central Baltic Sea. Number of samples are given above each group (*cod: p = .1e‐03; *flounder: p = 1.7e‐05).

The bulk δ^13^C values in cod and flounder from the central Baltic Sea were significantly lower than the values of conspecifics from the western Baltic Sea (*t*‐test, cod: *p* = .1e‐03; flounder: *p* = 1.7e‐05, Figure 4). However, flounder covered an overall larger δ^13^C range (−23.5‰ to −17.2‰) compared to cod (−21.7‰ to −19.3‰), both in the western and central Baltic Sea.

### 3.2 Environmental influence of benthic and pelagic food web structure

**FIGURE 5:**
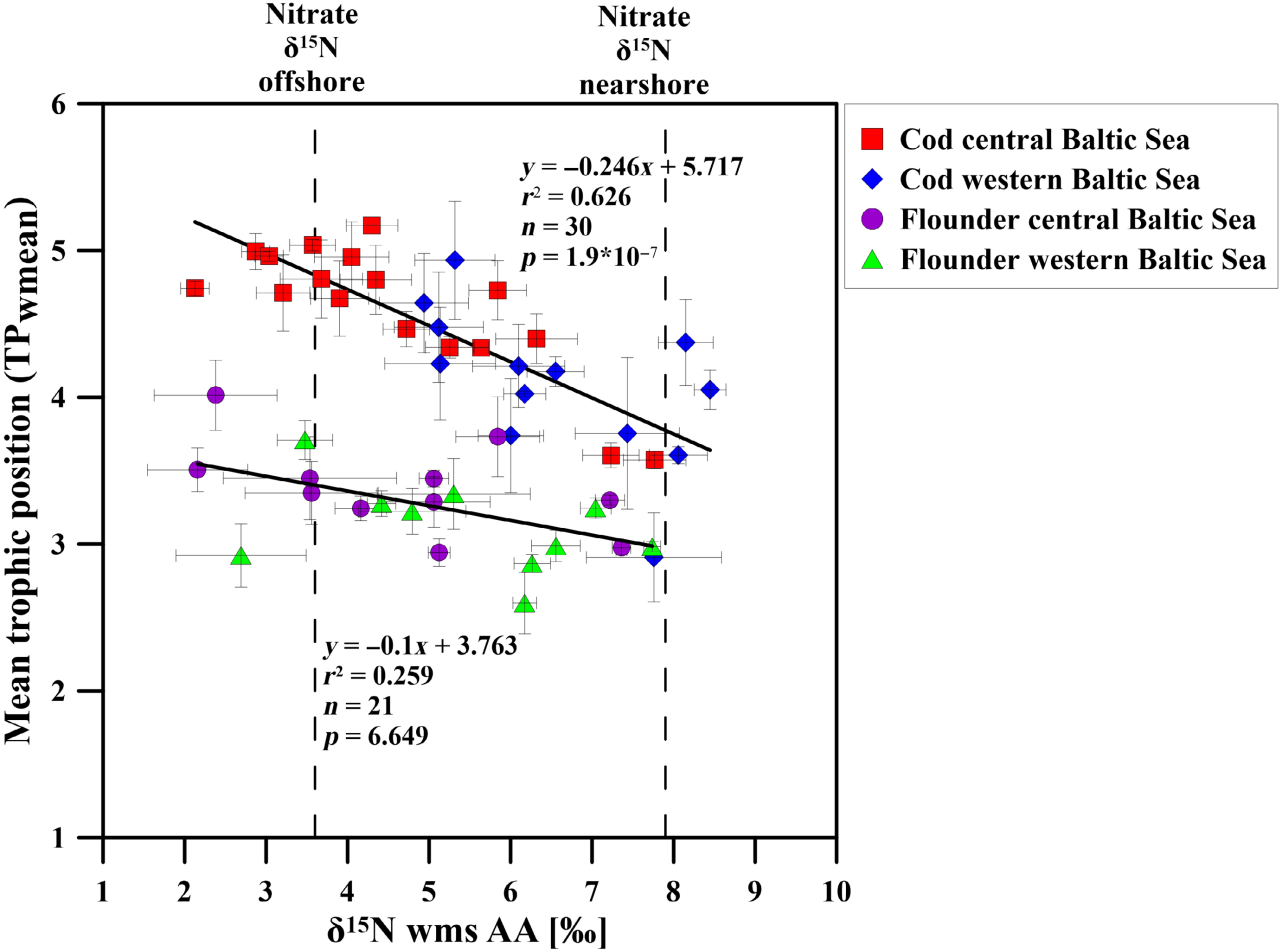
Mean trophic position (TP_wmean_) of cod and flounder as a function of the nitrogen source proxy δ^15^N_wms_ AA (δ^15^N values of the weighted mean source amino acids, in ‰) from the western (blue: cod, green: flounder) and central (red: cod, purple: flounder) Baltic Sea. Dashed lines: δ^15^N nitrate values influenced by N_2_ fixation (offshore central Baltic Sea) and by anthropogenic nitrogen (nearshore western/coastal Baltic Sea). Vertical/horizontal error bars indicate uncertainties in TP and the wms AA.

The N source measure δ^15^N of source AAs and the food web structure measure TP from the best fitting TP model were identified for both fish species and plotted against each other (Figure 5). Interestingly, δ^15^N values of the source AAs of cod varied strongly and indirectly with TP (*r* ^*2*^ = .63). This is in contrast to flounder, which displayed no significant relationship between TP and the N source measure (*r* ^*2*^ = .26).

Furthermore, a clear difference in the N source measure δ^15^N_wms_ was found in cod, with significantly lower δ^15^N_wms_ values of cod in the central than in the western Baltic Sea (*t*‐test, *p* = 7.4e‐06, Figure 5). Specifically, in the central Baltic, δ^15^N_wms_ values of cod ranged from 2.1‰ to 7.8‰, with a mean of 4.6‰ (SD ± 1.5, *n* = 17). For cod in the western Baltic Sea, the δ^15^N_wms_ values ranged from 4.9‰ to 8.4‰, with a mean of 6.8‰ (SD ± 1.3, *n* = 13). The δ^15^N_wms_ values of cod from the central Baltic closely resembled the δ^15^N endmember values for food webs with major N_2_ fixation (nitrate δ^15^N offshore, Figure 5), while in the western Baltic they reflected major anthropogenic N inputs (nitrate δ^15^N nearshore, Figure 5).

The difference in the N source measure was accompanied by a clear difference in the TP of cod with significantly higher TP values for cod in the central Baltic Sea than in the western Baltic Sea (*t*‐test, *p* = .004, Figure 5). The mean TP for cod from the western Baltic Sea was 4.1 ± 0.5 (*n* = 13), which was, as calibrated, very close to the SIA‐based western Baltic TP mean of 4.1 ± 0.2 (Deutsch & Berth, 2006; Mittermayr et al., 2014; Mohm, 2014, 2018) and also congruent to the global mean TP of cod (Froese & Pauly, 2022), but with a standard deviation twice as high as previously reported. Cod from the western Baltic Sea thus showed no impact of trophic lengthening but a higher plasticity in the TP value than the global mean. In strong contrast, the mean TP of cod from the central Baltic Sea was much higher at 4.6 ± 0.4 (*n* = 17) and thus clearly above the global mean of 4.1 ± 0.2 (Froese & Pauly, 2022). In summary, the TP estimates robustly showed that the mean TP of cod from the central Baltic Sea was significantly 0.5 TP units higher than the TP of cod from the western Baltic Sea.

In flounder, differences in the N source measure δ^15^N_wms_ between the two areas did not largely affect the TP. Interestingly, the δ^15^N_wms_ values in flounder varied widely and had a similar range (Figure 5), both in the western Baltic (range: 2.7–7.7‰; mean: 5.6 ‰ ± 1.5 ‰, *n* = 10) and in the central Baltic (range: 2.2–7.4‰; mean: 4.5‰ ± 1.8‰, *n* = 11). Thus, unlike cod, no direct assignment of individual flounder to the two areas was possible based on the N source measure δ^15^N_wms_. The TP of flounder also did not significantly differ between areas (*t*‐test, *p* = .06) and the mean TP value of 3.1 ± 0.3 was similar to the western Baltic mean TP value of flounder (Froese & Pauly, 2022; Mittermayr et al., 2014; Mohm, 2014, 2018).

**FIGURE 6:**
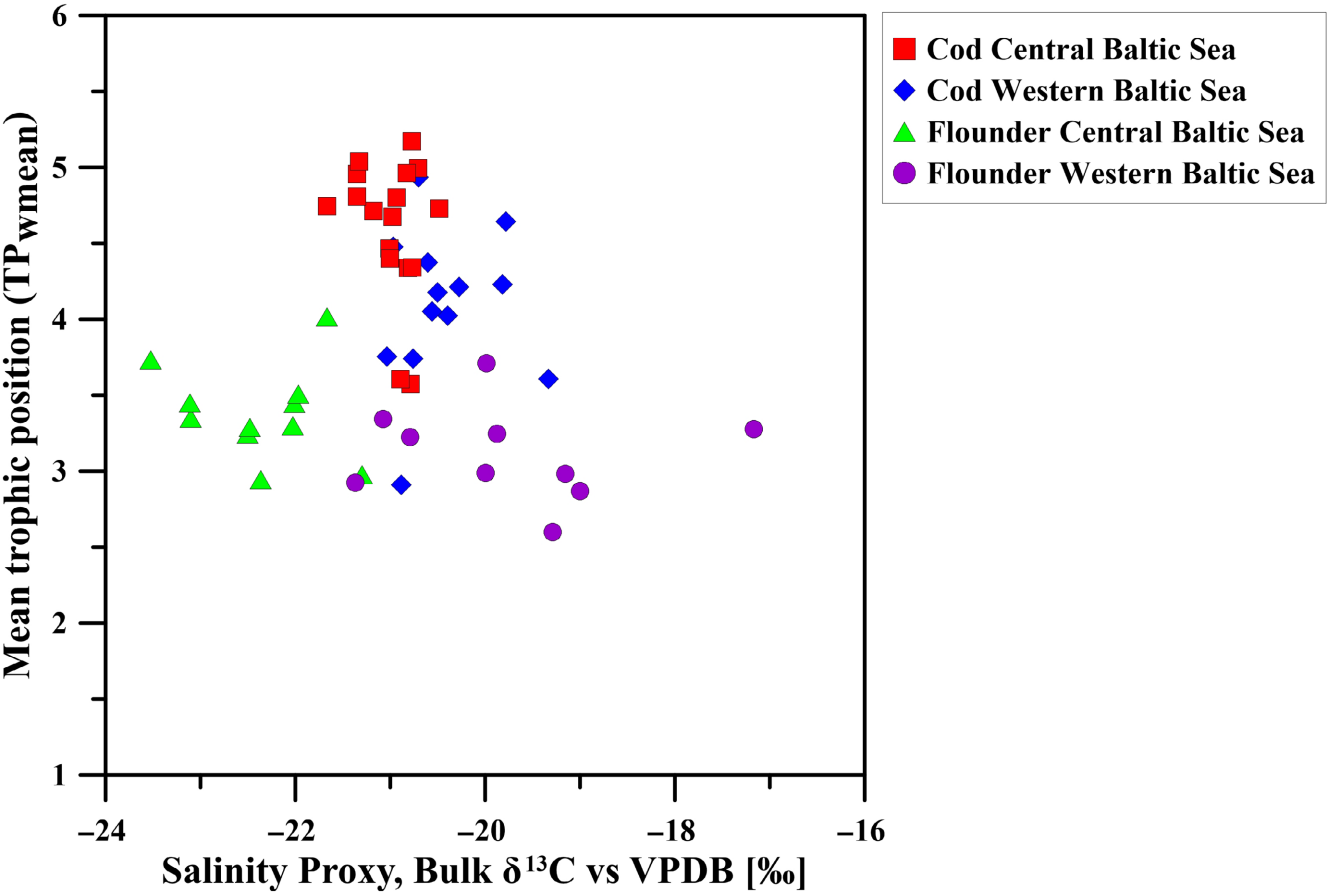
Mean trophic position (TP_wmean_) of cod and flounder as a function of bulk δ^13^C (in ‰) from the western (blue: cod, green: flounder) and central Baltic Sea (red: cod, purple: flounder).

A difference between flounder from the western and the central Baltic Sea only emerged when TP was plotted against the salinity proxy δ^13^C (Figure 6).

## 4 DISCUSSION

The recent quantification of trophic lengthening in mesozooplankton triggered by FNCs from the central Baltic Sea revealed a potentially important process of energy loss for commercial fish production, but the complexity and long turnover times of adult fish pose significant challenges for quantifying any impact of FNCs on their TP. We applied TP models based on empirical amino acid nitrogen stable isotope data to determine the N source and TP of individual fish from two Baltic Sea areas differently impacted by FNCs. Our data provide the first direct measurements of the N source and the TP in flounder and cod showing that trophic lengthening triggered by FNCs causes a significant increase in the TP of cod in the central Baltic Sea, while flounder, although caught in the same trawl hauls, is not affected.

### 4.1 New nitrogen source and role of export production for fish

The majority of cod caught in the Mecklenburg Bay had clearly relied on the isotopically heavier, shallow‐water food web of the western Baltic with anthropogenic N inputs and high δ^15^N values in thermocline nitrate. In contrast, the majority of cod caught in the Arkona and Bornholm Basins were linked to the isotopically lighter pelagic food web of the central Baltic with new N inputs from a thermocline nitrate pool that is rather impacted by diazotroph N.

Different to thermocline nitrate, diazotroph nitrogen per se was no important N source for adult cod in the central Baltic Sea. Here we define diazotroph N as inorganic and organic N species that directly originate from the process of N_2_ fixation in cyanobacteria. Diazotroph N can have the molecular structure of inorganic N like ammonium or nitrate as well as of organic N forms like amino acids. Diazotroph N in any molecular form has in common that its isotopic δ^15^N signature is 0‰ or lower as in case of diazotroph phenylalanine, which has a δ^15^N‐Phe of −3.6‰ in N_2_‐fixing marine cyanobacteria (Eglite et al., 2018; McClelland et al., 2003). In Figure 5, only four out of 17 cod individuals from the central Baltic Sea had δ^15^N_wms_ values somewhat below 3.6‰ pointing to a weak share of diazotroph N in their tissues, while the majority had δ^15^N_wms_ values close to the endmember value of 3.6‰ as for thermocline nitrate in the central Baltic Sea (Korth et al., 2014). Thus, no imprint of diazotroph nitrogen but of thermocline nitrate originating from the remineralization and nitrification of compounds originating from N_2_‐fixing cyanobacterial biomass (Korth et al., 2014) was found in cod from the central Baltic Sea. Finding the temporally integrated isotope signal of thermocline nitrate impacted by diazotroph N rather than of diazotroph N per se in the animals is not surprising. The share of diazotroph N in cod juveniles and larvae may be higher, but cod individuals analyzed in this study were older than 1 year and must have experienced multiple spring and summer blooms based on both, diazotroph nitrogen and thermocline nitrate originating from diazotroph N, in their lifetime.

In summary, the adult cod from the central Baltic Sea that were analyzed in this study showed no significant imprint of diazotroph nitrogen in their tissues but rather of thermocline nitrate originating from diazotroph nitrogen. Future CSIA studies have to show, how much diazotroph nitrogen gets incorporated by cod larvae and juveniles.

The lowest and highest values δ^15^N_wms_ occurring in both groups of cod suggested that some cod from the central Baltic Sea had spent time feeding near the coast and thus relied on a diet from the isotopically heavier nearshore food web with less N_2_ fixation but more eutrophication impacts on N sources (Korth et al., 2014; Voss et al., 2005). This can be the case in the Pomeranian Bay and other nearshore areas along the southern Baltic coast as suggested by recent recaptures of cod (Hüssy et al., 2020). Similarly, some cod caught in the western Baltic may have been exposed to FNC‐influenced isotopically lighter food web components which could be due to eastern Baltic cod foraging in areas normally inhabited by western Baltic cod (e.g., McQueen et al., 2019). In order to reduce uncertainty originating from a lack of individual stock assignment in an area affected by stock mixing, genetic analyses may be considered in the future and cod should preferentially be sampled further east of Bornholm. This could facilitate that mainly those cod will be analyzed that are part of the pelagic food web of the central Baltic Sea. A future simultaneous stomach content analysis of the sampled animals could also help to access the role of benthic and pelagic food in the diet of the sampled animals, which could influence the TP values.

Isotope mixing of thermocline nitrate with the isotopic imprint of either anthropogenic nitrogen or diazotroph nitrogen can account for the major trends in cod δ^15^N source AA values, but not in flounder, which showed a wide range of N source proxy data for both areas. In flounder, transient processes like isotopic fractionation associated with the uptake of nitrate by phytoplankton must also be considered to understand the δ^15^N_wms_ values. The wide range of flounder δ^15^N_wms_ values can be explain by the interaction of nitrate uptake by phytoplankton and export production. Export production is the amount of organic material produced in the ocean by new production that is not recycled (re‐mineralized) before sinking within a few hours to days (depending on water depth) from the euphotic zone to the bottom, where it becomes available to the benthic food web.

Isotope theory specific for nitrate‐based phytoplankton blooms predicts that during new production in phytoplankton enzymatic processes can produce different patterns of δ^15^N variation over time as the initial substrate pool of nitrate is consumed, for example, during spring bloom or when nitrate is injected into a system, for example by a river plume or upwelling (Montoya, 2007). If sufficient nitrate is still present at the beginning of a bloom, the lighter ^14^N nitrate is preferentially taken up, resulting in a 5–10‰ lower δ^15^N value in phytoplankton compared to the δ^15^N in nitrate (Montoya & McCarthy, 1995; Waser et al., 1998). As the bloom progresses and the phytoplankton takes up ^14^N‐nitrate with priority, the δ^15^N of the residual nitrate increases, which in turn leads to an increase in the δ^15^N of the developing seston. If there is little sedimentation of seston during a short bloom as part of export production, or if little phytoplankton is grazed by herbivores such as copepods, the δ^15^N of seston converges against the δ^15^N of the initial nitrate pool available for growth. If significant losses occur due to sedimentation or grazing, the current ^15^N signal from the phytoplankton will be found in the sinking seston and in the seston‐filtering macrozoobenthos, such as bivalves, or in the mesozooplankton. If these loss processes are high, the δ^15^N of seston in the euphotic zone can exceed and overshoot the initial δ^15^N of nitrate. Thus, the isotopic perturbation associated with a bloom can propagate to both, the pelagic and benthic food webs, potentially producing both higher and lower isotopic signals in particles relative to the initial δ^15^N in nitrate. Macrozoobenthos nitrogen and zooplankton nitrogen are converted more slowly than seston, so the magnitude of isotopic perturbation decreases with TP and the temporal pattern of δ^15^N fluctuations may be offset between trophic levels. If either of these disturbance signals in the phytoplankton biomass persists long enough relative to the turnover times of the macrozoobenthos or zooplankton biomass, these nitrate fractionation signals will propagate through the food web to the bivalves and zooplankton as well as higher trophic levels like fish. The extent of this signal propagation, in turn, provides a qualitative measure of the progression of the fractionation process in the nitrate pool, reflecting the time course of the energy flow characterizing the benthic or pelagic communities.

Applying these concepts to our data set, we might expect to see both low and high δ^15^N_wms_ readings in fish reflecting different stages of nitrate uptake at sites with large nitrate inputs. Indeed, we found very low δ^15^N_wms_ values in flounder at sites with high nitrate availability in the western Baltic Sea, but not in cod (Figure 5). Because δ^15^N_wms_ values below the nitrate value of 7.9‰ may originate from thermocline nitrate originating from diazotroph biomass, by fractionation in the early stages of isotopically heavy nitrate consumption, or from recycling (Montoya et al., 1992, 2002), we cannot fully elucidate the origin of these low δ^15^N_wms_ flounder signatures. That being said, the contextual clues of high nitrate concentrations and low δ^15^N_wms_ in flounder without corresponding low δ^15^N_wms_ in cod points to a transient process related to e uptake of isotopically heavy nitrate as the primary cause. This assumption is strengthened by the fact that also in the central Baltic Sea, where in contrast to the cod, very high δ^15^N_wms_ values were found in the flounder in addition to the low δ^15^N_wms_ values, which can be explained by isotope fractionation signals during nitrate uptake that has been transferred into flounder, but not into cod.

**FIGURE 7:**
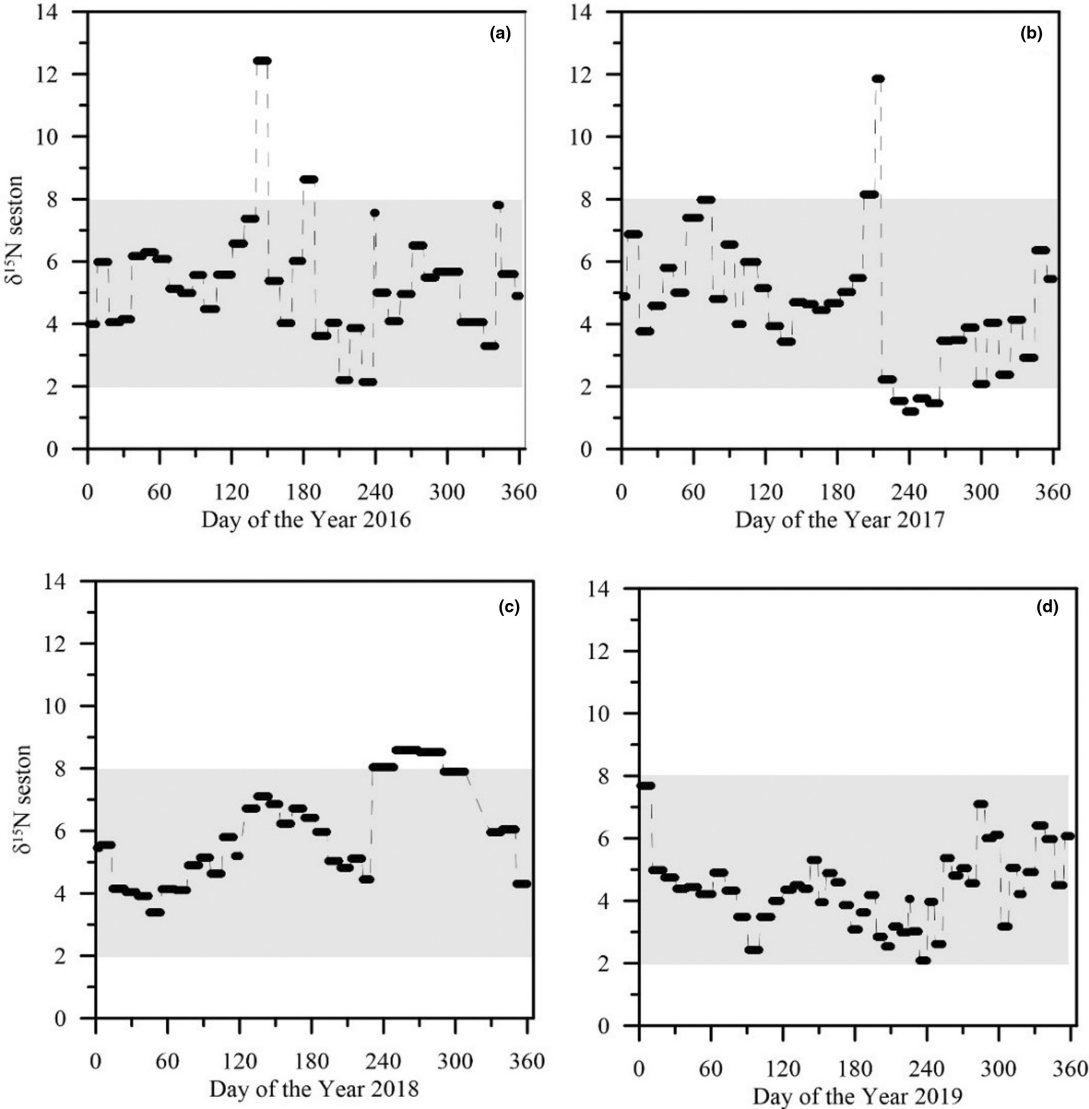
Isotopic signature of total nitrogen (δ^15^N in ‰) in seston from a sediment trap at 35 m depth in the central Arkona Basin in 2016–2019 (Wasmund et al., 2017, 2018, 2019; Zettler et al., 2020). Shaded in gray is the δ^15^N_wms_ value range between 2–8 ‰ found in flounder (see Figure 5).

This assumption is supported by δ^15^N values of total nitrogen in seston from sediment trap samples collected at 35 m depth in the Arkona Basin (Figure 7).

Specifically, the 2‐ to 4‐year‐old flounder caught in 2019 and 2020 and measured here have the same range of δ^15^N_wms_ values as export production in the form of δ^15^N of seston from the Arkona Basin in their years of growth from 2016–2019. Except for a few outliers in seston, δ^15^N values in both, the seston and flounder varied between 2–8‰ (Figures 5 and 7).

In summary, since flounder mainly feed on seston incorporating bivalves, the most likely explanation for the wide δ^15^N_wms_ ranges in the flounder from both areas is that their δ^15^N_wms_ values, just like the δ^15^N of seston in the sediment trap samples, reflect isotopic fractionation in export production. Since the cod follows a completely different foraging strategy and feeds on considerably higher TPs than the flounder, it receives a smoothed N source signal without perturbation from isotope fractionation. The δ^15^Nwms values in cod are rather characterized by the isotopic mixing of thermocline nitrate, which is formed from anthropogenic nitrogen in the western Baltic Sea and from diazotroph nitrogen in the central Baltic Sea.

### 4.2 Cyanobacteria and trophic lengthening in the pelagic but not the benthic food web

Cod in the central Baltic Sea fed on organisms from a significantly higher TP than cod in the western Baltic. The difference corresponded to 0.5 TP units, if all cod individuals from the central Baltic Sea were included. This is a conservative estimation given that it also includes animals with δ^15^N_wms_ values above the δ^15^N endmember value of 3.6 ± 1.0‰ for thermocline nitrate and surface sediment particles from the central Baltic Sea (Korth et al., 2014) that likely are migrators to coastal waters. Excluding any potential migrators, the difference in TP between cod from the western and central Baltic Sea increases to 0.8 TP units.

**FIGURE 8:**
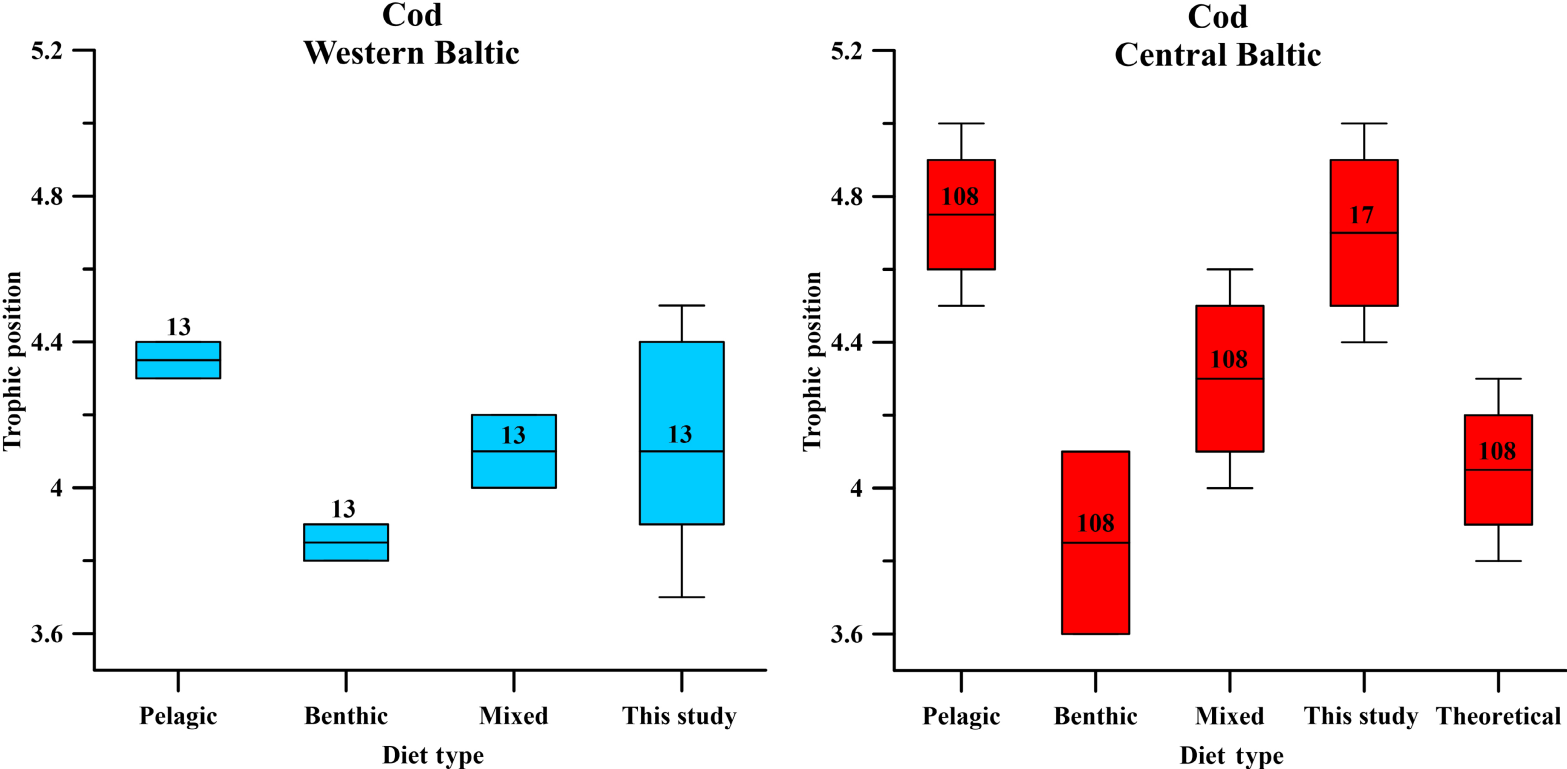
Boxplot of the SIA‐based TP values of cod from the western and central Baltic Sea calculated after Post (2002) from literature data (Western Baltic: Deutsch & Berth, 2006; Mohm, 2014, 2018, Eastern Baltic: Deutsch & Berth, 2006; Mohm, 2014, 2018) for different assumed food preferences (pelagic, benthic, mixed = mix of both) and of CSIA‐based TP values (Table 2). For the central Baltic Sea additional theoretical TP values for cod with a pelagic food preference were calculated excluding the effect of trophic lengthening on the mesozooplankton compartment (TP of primary consumer of 2.0 rather than 2.7, Table 2).The high TPs in cod from the central Baltic Sea from this study only can be explained by a preferential pelagic diet including the effect of trophic lengthening. See text for more details.

Interestingly, other studies have also found increased TP values in the food web of the central Baltic Sea (Kiljunen et al., 2020) compared to TP values for the same species/compartment from the food web of the western Baltic Sea (Mittermayr et al., 2014, Table 2). The higher TP values in herring and sprat from the central Baltic Sea (Kiljunen et al., 2020) are particularly interesting here, as they are the missing link between zooplankton and cod. The higher TPs in cod from the central compared to the western Baltic Sea cannot be explained by differences in prey alone. We calculated the TP of cod from the western and central Baltic Sea based on bulk δ^15^N values from literature and estimated their TP for different types of diet to compare them with the CSIA‐based TPs of cod (Table 2, Figure 8).

Although cod from the central Baltic Sea feed in some parts on benthic food such as *Saduria entomon* (Kulatska et al., 2019), purely benthic or mixed benthic and pelagic diet of cod from the central Baltic Sea would lead to similar low TPs as in cod from the western Baltic Sea (Figure 8). Further, a purely pelagic diet of western cod would not lead to elevated TPs as we found for cod from the central Baltic Sea (Figure 8). Ruling out any trophic lengthening effect by assuming a TP of 2.0 rather than of 2.7 for zooplankton (called theoretical diet type in Figure 8) would also lower the TP of cod in the central Baltic Sea down to western Baltic Sea TP levels. Figure 8 reveals that only the SIA‐based TP calculation for cod including both, a TP of 2.7 for zooplankton and a high proportion of pelagic herring and sprat in the diet, reaches the high TP values in cod as estimated from the CSIA‐based TP approach. This shows that trophic lengthening has an important influence on cod from the central Baltic Sea. Yet, without a small share of benthic diet and/or times of growth without trophic lengthening in the pelagic food web (e.g., during the spring bloom of diatoms), cod from the central Baltic Sea would probably have even higher TP values than those found in this study.

**FIGURE 9:**
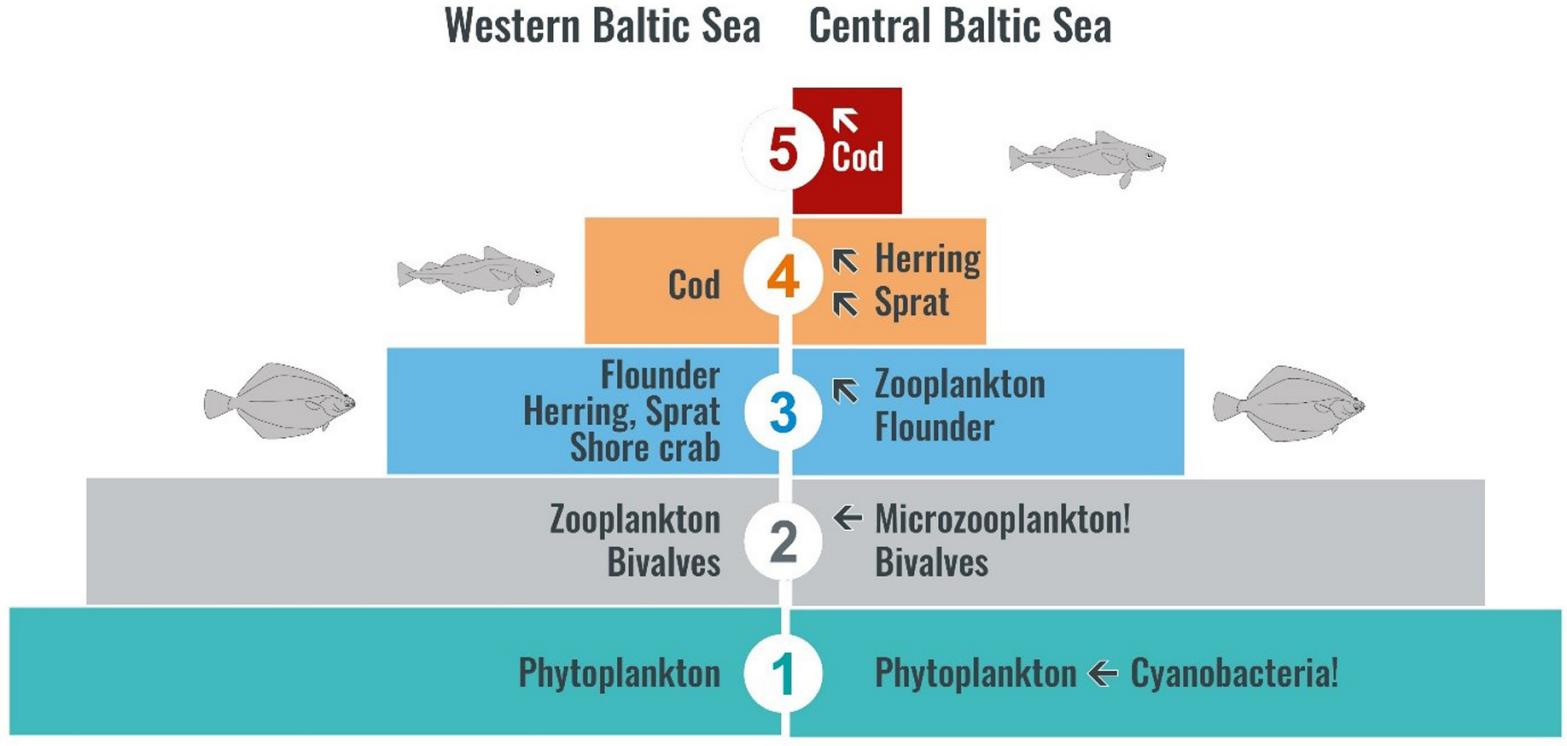
Trophic pyramid including the trophic positions (TPs) of key organisms of the pelagic and benthic food webs in the western and central Baltic Sea. Only the pelagic food web in the central Baltic is affected by trophic lengthening due to the growth of filamentous, N_2_‐fixing cyanobacteria and associated microzooplankton (**←**!), which together cause increased TPs (**↖**!) up to the level of cod. The benthic food web including flounder is not affected by trophic lengthening in neither area.

Higher TPs of cod from the central Baltic Sea than of cod in the western Baltic robustly demonstrate the long‐lasting effect of a tropically longer food web affected by an FNCs induced influence of the microbial system. Given the strong correlation of TP with the N source measure δ^15^N_wms_ in cod (Figure 5), the underlying mechanism must be related to the dominance of FNCs, which are largely unpalatable and thus not subject to direct grazing (Loick‐Wilde et al., 2012; Motwani et al., 2018; Wannicke et al., 2013). Instead, a functionally more complex food base of protists, flagellates, ciliates, bacteria, viruses, and other microorganisms gains a more important influence with the unpalatable cyanobacteria (Eigemann & Schulz‐Vogt, 2019; Sheridan et al., 2002). However, this also leads to a feedback loop in late summer, as the microbial system mediates the nutrients of the now dying and sinking cyanobacteria back into the system, which can support new phytoplankton growth. Also some of these organisms, namely microzooplankton like flagellates and ciliates, form now an additional trophic level in the pelagic food web when they serve as a dietary source for otherwise mainly herbivorous or maximally omnivorous mesozooplankton, which thus becomes carnivorous (Loick‐Wilde et al., 2019). In the central Baltic Sea, this is especially true for *Temora longicornis*, a copepod species that predominantly feeds on flagellates and ciliates during cyanobacterial blooms and reaches a TP of 3.0 (Dutz et al., 2012; Eglite et al., 2018; Loick‐Wilde et al., 2019). The food chain is lengthened accordingly and the effect is transferred to higher trophic levels. Interestingly, sprat and herring, which are widespread in the Baltic Sea and which in case of sprat mainly feed on *T. longicornis* (Bernreuther et al., 2018; Möllmann et al., 2004), also have higher TPs in the central Baltic Sea (Kiljunen et al., 2020) compared to the western Baltic Sea (Table 2). Ultimately, sprat and herring is eaten by cod in the central/eastern Baltic Sea, which increases the TP of cod from ~4.0 to near 5.0 as shown in this study (Figure 9).

This clearly shows how trophic lengthening at the base of the pelagic food web can change the TP of its higher secondary consumers. The cyanobacteria are the trigger of this effect, but not the general effector. The general effector is the microbial system including microzooplankton, which can modulate (cause or negate) a trophic lengthening through increasing or decreasing importance within flows and feedback loops in the food web. The microbial structure exists in every aquatic system and changes in the trophic flow through it (e.g., caused by FNCs) can cause trophic lengthening. In the central Baltic Sea, the trigger for massive microbial systems are FNCs. However, massive microbial systems can also be caused by other factors such as eutrophication (Dähnke et al., 2008) or oxygen minimum zones (Fernández‐Urruzola et al., 2023).

**FIGURE 10:**
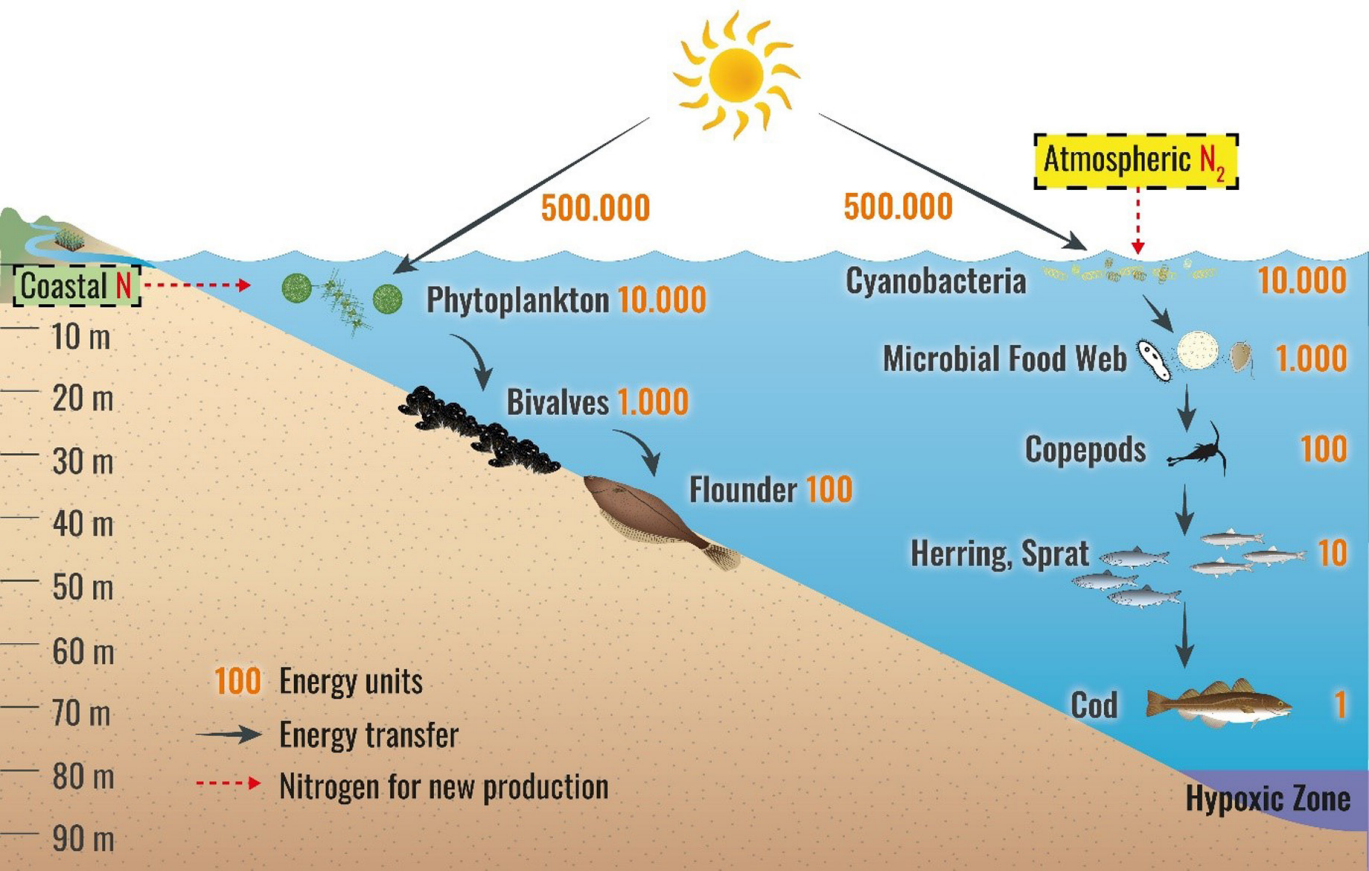
Conceptual view of the energy transfer to benthic and pelagic food webs in the central Baltic Sea including the process of trophic lengthening in the pelagic food web due to an FNC‐triggered massive microbial food web. Efficiency of energy to phytoplankton due to low conversion efficiencies in photosystems (Melis, 2009). See text for more details.

In flounder, there were no significant differences or trends in the mean TPs between the two areas and the scatter of the individual TPs of flounder was lower than in cod. This suggests that flounder has a narrower diet composition and is decoupled from trophic lengthening in the pelagic food web. Mesozooplankton is the central pivot in pelagic food webs which enables the transfer of mass and energy from the phytoplankton to the higher trophic levels including cod. For flounder, mesozooplankton is of minor importance because flounder almost exclusively feed on bivalves (Haase et al., 2020). Bivalves closely reflect the δ^15^N signal in seston from exported new production and transfer it into the δ^15^N_wms_ of flounder. This highlights a major difference in the functioning of the benthic compared to the pelagic food web of the central Baltic Sea: Without the transfer of mass and energy via mesozooplankton, flounder is unaffected by the trophic lengthening that disrupts the energy transfer in the pelagic food web (Figure 10) and leads to a higher TP in cod (Figure 9).

### 4.3 FNC‐caused disruption of the pelagic but not the benthic food webs

Trophic lengthening in a food web inevitably leads to losses of thermal and chemical energy at higher trophic levels. Following the second law of thermodynamics, a pyramid of energy can never be inverted since it accounts for the turnover rate of the organisms with energy loss as heat and waste products in all metabolic processes involved (Reichle, 2023). However, in contrast to heat and waste product losses, the associated increases in TP can now be measured directly in organisms using CSIA.

Given a trophic efficiency of about 10%, trophic lengthening results in a massive decrease in biomass production, meaning that instead of the previous 0.1%, only 0.01% of the energy and mass from new production reaches the cod stock in the central Baltic Sea (Figure 10).

In other words, assuming an input of new nitrogen via N_2_ fixation of 370,000–926,000 kt N year^−1^ in the Baltic Sea (Voss et al., 2005), only 37.0–92.6 kt N year^−1^ instead of 370–926 kt N year^−1^ of nitrogen of the annual new production would reach the end of the food web and be incorporated in cod and other predators at a TP of 5.0. FNC‐triggered an enormous increase in production at times and the production of cyanobacteria is closely coupled to the rest of the food web. However, because there is almost no direct grazing of FNCs, the mass and energy is transported mainly indirectly to zooplankton via the microbial food web including microzooplankton with inevitable large losses of mass and energy as heat and waste products.

In contrast to cod, the TP flounder was not affected, thus the position of flounder in a trophic pyramid (Figure 9) and the energy transfer (Figure 10) is not changed by FNC‐caused trophic lengthening. Therefore, the missing or massive effect of trophic lengthening on the benthic or pelagic food web, respectively, could explain the stable spawning stock biomass (SSB) of flounder (ICES, 2023) and the historic low SSB of cod, respectively, in the central Baltic Sea (Eero et al., 2023).

### 4.4 Past and future impact of trophic lengthening on eastern Baltic cod

FNC‐triggered trophic lengthening does not seem to be a phenomenon of the 21st century. FNC blooms are closely linked to eutrophic conditions, namely the supply of phosphorus, which limits N_2_ fixation in the Baltic Sea (Conley, 2012; Voss et al., 2011). Anthropogenic eutrophication is not a recent stressor in the Baltic Sea (Andersen et al., 2017; Murray et al., 2019). Analyses of sediment cores revealed that massive blooms of *Nodularia spumigena* and *Aphanizomenon flos‐aquae* already occurred at the beginning of the 20th century in the central Baltic Sea (Kaiser et al., 2020). A potential lengthening of the food web due to unpalatable primary producers and accompanied detrimental effects for cod production in this environment may, therefore, have played a role even before the historic SSB peak of the eastern Baltic cod (EBC) stock in the early 1980s (Eero et al., 2023) since the SSB of EBC also showed remarkable lows in the 1960/70s. Interestingly, the SSB peak of the EBC in the early 1980s coincided with lower sea surface temperatures and a lower frequency of FNC accumulations (Kahru et al., 2020; Kaiser et al., 2020). The SSB decline since the 1990s was accompanied by a rapid increase in the bloom frequency of FNCs and a rise in sea surface temperatures. Moreover, the historical decline in EBC SSB between the 1980s and 1990s matches the period when an increase of the EBC TP by 0.4–0.7 units took place and which stayed high in EBC ever since. During this period a major biological regime shift took place in the central Baltic Sea (Alheit, 2009; Alheit et al., 2005; Möllmann et al., 2008, 2009). One so far unconsidered and unaccounted underlying mechanism for this change could be related to the FNC blooms, which lead to a trophic lengthening of the food chain and a change in the energy transfer (Figure 8). This is supported by biomass data of copepods, which show that *Pseudocalanus acuspes* was gradually replaced by, for example, *Temora longicornis*, a copepod that has been shown to feed at higher TPs, that is, it preys upon ciliates and flagellates (Dutz et al., 2010, 2012). CSIA‐based TP estimates of central Baltic herring and Baltic sprat are missing but the high SIA‐based TP estimates (Kiljunen et al., 2020; Table 2) and much lower level in mean weight‐at‐age of herring and sprat since the 1990s (ICES, 2023) strongly suggest that these small, zooplanktivouros pelagics were also affected by trophic lengthening. This lengthening of the pelagic food web from the mid 1980s was apparently strong enough to eventually reach the EBC stock and negatively affect stock productivity.

## 5 OUTLOOK

The Baltic Sea serves as an exemplary model ecosystem that provides significant insights into how food webs and energy flows can change due to FNC events. A progressively carnivorous mesozooplankton compartment fosters greater inefficiency in energy transfer to higher trophic levels compared to a mainly herbivorous zooplankton community. FNC‐triggered increase of the influence of the microbial system that induces trophic lengthening at the base of the food web can lead to massive losses in productivity of predatory species. It is a testable hypothesis that the observed trophic lengthening up to the mesopredators like cod in the Baltic Sea also takes place in *Trichodesmium*‐affected coastal, marine ecosystems.

CSIA is an objective method to unambiguously determine the TP of key species. Monitoring the deviations of the TPs from the global mean values of aquatic species that show strong biomass fluctuations (in the central Baltic for example herring, sprat and cod) would allow to identify fundamental changes in ecosystems, the recovery potential of FNC‐affected fish stocks and can, therefore, provide useful information for resource management.

## AUTHOR CONTRIBUTIONS

**Markus Steinkopf:** Conceptualization (equal); investigation (equal); methodology (equal); visualization (equal); writing – original draft (equal). **Uwe Krumme:** Conceptualization (equal); investigation (equal); supervision (equal); visualization (equal); writing – review and editing (equal). **Detlef Schulz‐Bull:** Methodology (equal); supervision (equal); writing – review and editing (equal). **Dirk Wodarg:** Methodology (equal); writing – review and editing (equal). **Natalie Loick‐Wilde:** Conceptualization (equal); investigation (equal); methodology (equal); supervision (equal); visualization (equal); writing – review and editing (equal).

## FUNDING INFORMATION

This study was supported by the BluEs project, funded by the BMBF (grant number FKZ 03F0864A).

## CONFLICT OF INTEREST STATEMENT

All other authors declare they have no competing interests.

## Supporting information


Table S1.

## Data Availability

Raw data of elemental and stable isotopes analyses are available at Dryad through DOI: 10.5061/dryad.12jm63z48.
