# Supplementary material for: Trophic lengthening triggered by filamentous, N2 ‐fixing cyanobacteria disrupts pelagic but not benthic food webs in a large estuarine ecosystem
Source: Ecol Evol. 2024 Feb 20;14(2):e11048. doi: 10.1002/ece3.11048 (PMC10877452; doi:10.1002/ece3.11048)
Supplement: Supplementary file 1 — Table S1. [file ECE3-14-e11048-s001.docx]

**Appendix**

Table A1: Individual fish sampled for this study. By species, station, depth stratum, sex (f = female, m = male, n.d. = no data), size (cm), ICES-Subdivision (SD), areas in the Baltic Sea (BS = Belt Sea, AB = Arkona Basin, BB = Bornholm Basin), cruise (SB = FRV “Solea”, C = FRV “Clupea”) and month/year.

| **Species** | **Station** | **Depth [m]** | **Sex** | **Size [cm]** | **ICES-SD** | **Area** | **Cruise** | **Month/Year** |
| --- | --- | --- | --- | --- | --- | --- | --- | --- |
| Cod | 1 | 20-30 | f | 35 | 22 | BS | SB774 | Feb 20 |
| Cod | 2 | 20-30 | f | 20 | 22 | BS | SB774 | Feb 20 |
| Cod | 4 | 10-20 | m | 33 | 22 | BS | SB774 | Feb 20 |
| Cod | 4 | 10-20 | f | 29 | 22 | BS | SB774 | Feb 20 |
| Cod | 4 | 10-20 | m | 34 | 22 | BS | SB774 | Feb 20 |
| Cod | 4 | 10-20 | m | 35 | 22 | BS | SB774 | Feb 20 |
| Cod | 4 | 10-20 | f | 34 | 22 | BS | SB774 | Feb 20 |
| Cod | 4 | 10-20 | f | 32 | 22 | BS | SB774 | Feb 20 |
| Cod | 5 | 20-30 | m | 20-30 | 22 | BS | C341 | Jan 20 |
| Cod | 6 | 20-30 | m | 20-30 | 22 | BS | C341 | Jan 20 |
| Cod | 8 | 10-20 | f | 20-30 | 22 | BS | C341 | Jan 20 |
| Cod | 8 | 10-20 | f | 20-30 | 22 | BS | C341 | Jan 20 |
| Cod | 8 | 10-20 | m | 20-30 | 22 | BS | C341 | Jan 20 |
| Cod | 10 | 10-20 | n.d. | 38 | 24 | AB | SB759 | Feb 19 |
| Cod | 11 | 50-60 | n.d. | 23 | 24 | AB | SB759 | Feb 19 |
| Cod | 11 | 50-60 | n.d. | 32 | 24 | AB | SB759 | Feb 19 |
| Cod | 11 | 50-60 | n.d. | 34 | 24 | AB | SB759 | Feb 19 |
| Cod | 11 | 50-60 | n.d. | 36 | 24 | AB | SB759 | Feb 19 |
| Cod | 11 | 50-60 | n.d. | 36 | 24 | AB | SB759 | Feb 19 |
| Cod | 12 | 60-70 | f | 36 | 25 | BB | SB773 | Feb 20 |
| Cod | 13 | 60-70 | n.d. | 29 | 25 | BB | SB759 | Feb 19 |
| Cod | 13 | 60-70 | n.d. | 29 | 25 | BB | SB759 | Feb 19 |
| Cod | 13 | 60-70 | n.d. | 35 | 25 | BB | SB759 | Feb 19 |
| Cod | 13 | 60-70 | n.d. | 36 | 25 | BB | SB759 | Feb 19 |
| Cod | 13 | 60-70 | n.d. | 32 | 25 | BB | SB759 | Feb 19 |
| Cod | 14 | 60-70 | m | 20-30 | 25 | BB | SB773 | Feb 20 |
| Cod | 14 | 60-70 | f | 20-30 | 25 | BB | SB773 | Feb 20 |
| Cod | 14 | 60-70 | m | 20-30 | 25 | BB | SB773 | Feb 20 |
| Cod | 15 | 60-70 | f | 20-30 | 25 | BB | SB773 | Feb 20 |
| Cod | 15 | 60-70 | m | 20-30 | 25 | BB | SB773 | Feb 20 |
| Flounder | 3 | 20-30 | f | 20-30 | 22 | BS | SB774 | Feb 20 |
| Flounder | 4 | 20-30 | f | 20-30 | 22 | BS | SB774 | Feb 20 |
| Flounder | 7 | 20-30 | n.d. | 20-30 | 22 | BS | C341 | Jan 20 |
| Flounder | 7 | 20-30 | n.d. | 20-30 | 22 | BS | C341 | Jan 20 |
| Flounder | 9 | 20-30 | n.d. | 20-30 | 22 | BS | C341 | Jan 20 |
| Flounder | 9 | 20-30 | n.d. | 20-30 | 22 | BS | C341 | Jan 20 |
| Flounder | 9 | 20-30 | n.d. | 20-30 | 22 | BS | C341 | Jan 20 |
| Flounder | 9 | 20-30 | n.d. | 20-30 | 22 | BS | C341 | Jan 20 |
| Flounder | 9 | 20-30 | n.d. | 20-30 | 22 | BS | C341 | Jan 20 |
| Flounder | 9 | 20-30 | n.d. | 20-30 | 22 | BS | C341 | Jan 20 |
| Flounder | 13 | 60-70 | n.d. | 20-30 | 24 | AB | SB759 | Feb 19 |
| Flounder | 13 | 60-70 | n.d. | 20-30 | 24 | AB | SB759 | Feb 19 |
| Flounder | 13 | 60-70 | n.d. | 20-30 | 24 | AB | SB759 | Feb 19 |
| Flounder | 13 | 60-70 | n.d. | 20-30 | 24 | AB | SB759 | Feb 19 |
| Flounder | 13 | 60-70 | n.d. | 20-30 | 24 | AB | SB759 | Feb 19 |
| Flounder | 14 | 20-30 | m | 20-30 | 25 | BB | SB773 | Feb 20 |
| Flounder | 14 | 20-30 | f | 20-30 | 25 | BB | SB773 | Feb 20 |
| Flounder | 14 | 20-30 | m | 20-30 | 25 | BB | SB773 | Feb 20 |
| Flounder | 14 | 20-30 | m | 20-30 | 25 | BB | SB773 | Feb 20 |
| Flounder | 14 | 20-30 | f | 20-30 | 25 | BB | SB773 | Feb 20 |
| Flounder | 14 | 20-30 | n.d. | 20-30 | 25 | BB | SB773 | Feb 20 |
